# Neutrophil extracellular traps enhance macrophage killing of bacterial pathogens

**DOI:** 10.1126/sciadv.abj2101

**Published:** 2021-09-10

**Authors:** Andrew J. Monteith, Jeanette M. Miller, C. Noel Maxwell, Walter J. Chazin, Eric P. Skaar

**Affiliations:** 1 Department of Pathology, Microbiology, and Immunology, Vanderbilt University Medical Center, Nashville, TN, USA.; 2 Department of Biochemistry, Vanderbilt University, Nashville, TN, USA.; 3 Center for Structural Biology, Vanderbilt University, Nashville, TN, USA.; 4 Department of Chemistry, Vanderbilt University, Nashville, TN, USA.; 5 Vanderbilt Institute for Infection, Immunology, and Inflammation, Vanderbilt University Medical Center, Nashville, TN, USA.

## Abstract

Neutrophils and macrophages are critical to the innate immune response, but cooperative mechanisms used by these cells to combat extracellular pathogens are not well understood. This study reveals that S100A9-deficient neutrophils produce higher levels of mitochondrial superoxide in response to *Staphylococcus aureus* and, as a result, form neutrophil extracellular traps (suicidal NETosis). Increased suicidal NETosis does not improve neutrophil killing of *S. aureus* in isolation but augments macrophage killing. NET formation enhances antibacterial activity by increasing phagocytosis by macrophages and by transferring neutrophil-specific antimicrobial peptides to them. Similar results were observed in response to other phylogenetically distinct bacterial pathogens including *Streptococcus pneumoniae* and *Pseudomonas aeruginosa*, implicating this as an immune defense mechanism that broadly enhances antibacterial activity. These results demonstrate that achieving maximal bactericidal activity through NET formation requires macrophages and that accelerated and more robust suicidal NETosis makes neutrophils adept at increasing antibacterial activity, especially when A9 deficient.

## Teaser

S100A9 regulates mitochondrial-mediated neutrophil extracellular trap formation, which augments macrophage bactericidal activity.

## INTRODUCTION

*Staphylococcus aureus* is a Gram-positive bacterium that causes a wide range of infections and is the leading cause of bacterial endocarditis (*1*) and second most frequent agent of bloodstream infections (*2*). Professional phagocytes, such as neutrophils and macrophages (Mφs), have an arsenal of antimicrobial processes that are critical to combating bacterial pathogens. During infection, phagocytosis of *S. aureus* by neutrophils initiates bacterial killing through the production of reactive oxygen species (ROS) by NADPH (nicotinamide adenine dinucleotide phosphate) oxidase (*3*–*5*) or fusing granules containing antimicrobial peptides (AMPs) to the phagosome (*6*, *7*). In addition, neutrophils combat extracellular pathogens by secreting neutrophil extracellular traps (NETs) consisting of DNA studded with AMPs through a process termed “NETosis.” Within 5 to 60 min of engaging *S. aureus*, vital NETosis results in the exocytosis of NETs leaving the membrane intact (*8*), while suicidal NETosis, which occurs after 2 to 4 hours, causes rupture of the cell membrane through the terminal release of NETs (*9*). *S. aureus* induces NETosis, but the antibacterial activity of NETs varies depending on the context, where NET formation is beneficial to the host during pneumonia (*10*) and systemic infections (*11*) but detrimental in combating biofilms (*12*). However, in vitro, the biological function of NETs in response to bacterial pathogens has only been assessed in isolation with neutrophils being the only cell type present, which does not reflect the full cellular complexity of the host-pathogen interface.

Calprotectin, a heterodimer formed by the S100A8 and S100A9 proteins (*13*, *14*), is enriched within neutrophils comprising approximately 50% of the cytoplasmic protein (*15*). During inflammation, calprotectin sequesters transition metals from pathogens (*16*, *17*) and influences immune cell function as a chemoattractant (*18*, *19*) and damage-associated molecular pattern (DAMP) (*20*, *21*). In addition, S100A9-deficient (A9^−/−^) mice, which lack calprotectin, exhibit increased *S. aureus* burdens in the kidney and liver (*16*, *17*) but lower burdens in the heart with increased survival (*22*) when systemically infected. This indicates that S100A9 may have alternative functions in regulating the immune response in an organ-specific manner.

Mφs are also critical to the innate immune response to *S. aureus* (*23*, *24*) and elicit most of their antimicrobial activity after internalizing the pathogen. Following phagocytosis, Mφs generate ROS and reactive nitrogen species (*25*, *26*), mobilize transition metals (*27*, *28*), and acidify the phagosome to activate hydrolytic enzymes (*29*, *30*) in an effort to intoxicate pathogens. Despite these antimicrobial functions, Mφs are not effective at eradicating internalized *S. aureus* in isolation (*23*, *31*, *32*).

Neutrophils can synergistically enhance the antibacterial function of Mφs. Phagocytosis of infected apoptotic neutrophils allows Mφs to acquire functionally active myeloperoxidase (MPO), lactoferrin, and defensins from neutrophil granules, thereby enhancing antimicrobial activity (*33*–*36*); however, this phenomenon has only been observed in response to intracellular bacterial pathogens. During staphylococcal infection, both neutrophils (*37*, *38*) and Mφs (*39*–*42*) aid in the formation and maintenance of abscesses, putting these cells in close proximity. Yet, most host-pathogen interactions studies involving *S. aureus* have been assessed using isolated immune cells or whole blood lacking mature Mφs. Defining how neutrophils and Mφs cooperatively combat *S. aureus* will help define an effective innate immune response to *S. aureus* and to provide insight into antibacterial strategies that could be broadly applied to other extracellular bacterial pathogens.

Here, we show that A9^−/−^ neutrophils produce higher levels of mitochondrial superoxide (O_2_^−^) in response to *S. aureus*, causing accelerated and robust suicidal NETosis and attenuated primary degranulation. This establishes intracellular S100A9 as a critical molecular rheostat in neutrophil function. Increased suicidal NETosis does not provide an advantage in neutrophil-mediated killing of bacterial pathogens in isolation but rather augments Mφ killing in response to multiple phylogenetically distinct pathogens, including *S. aureus*, *Streptococcus pneumoniae,* and *Pseudomonas aeruginosa*. NET formation increases the antibacterial activity of Mφs by facilitating their phagocytosis of bacteria and by transferring biologically active neutrophil-specific AMPs to Mφs. By using A9^−/−^ neutrophils as a tool to assess NET-related biology, these results demonstrate that NET formation acts as a conduit for neutrophils to enhance the antibacterial activity of Mφs. Furthermore, neutrophils with accelerated and more robust suicidal NETosis, such as A9^−/−^, are more adept at increasing the antibacterial activity of Mφs against multiple bacterial pathogens.

## RESULTS

### A9^−/−^ mice have increased protection from methicillin-resistant *S. aureus*

**Fig. 1.**
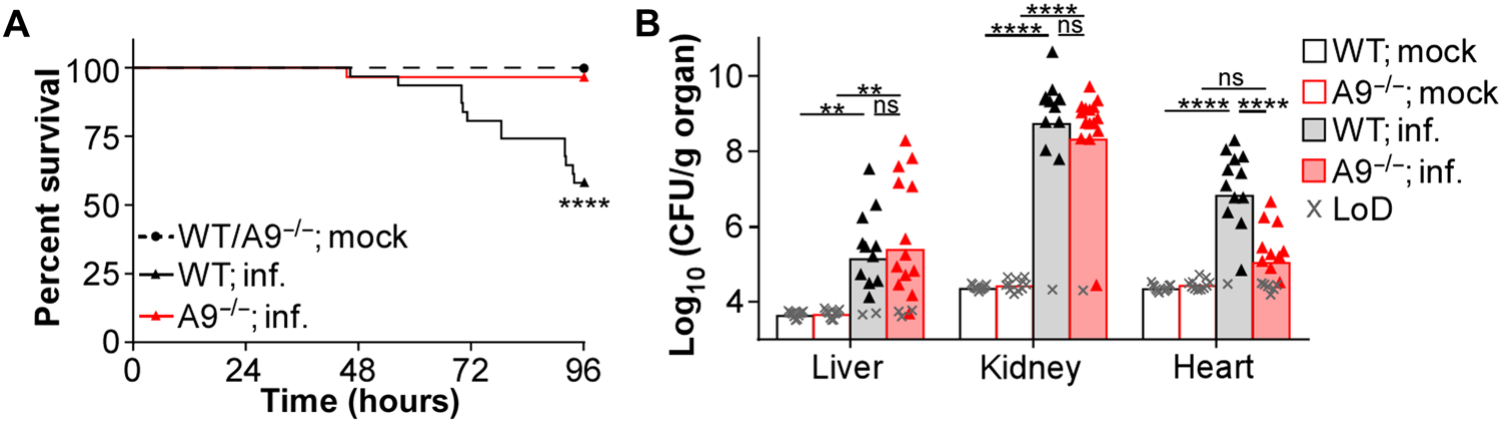
A9^−/−^ mice are more protected from systemic MRSA infection. Mice were systemically infected (inf.) with USA300 [colony-forming units (CFU) = 2 × 10^7^]. (**A**) During the infection, mouse survival was monitored. Each point represents the percentage of living mice (mock, *n* = 14, WT; inf., *n* = 31, A9^−/−^; inf., *n* = 29). (**B**) At 4 dpi, organs were homogenized and CFU was counted using spot plating [limit of detection (LoD)]. Each point represents a single mouse (mock, *n* = 11) (WT; inf., *n* = 13) (A9^−/−^; inf., *n* = 16). (A) Log-rank (Mantel-Cox) test or (B) two-way analysis of variance (ANOVA) with Tukey multiple comparisons test (**P ≤ 0.01 and ****P ≤ 0.0001; ns, not significant).

Previous systemic infections of A9^−/−^ mice used the *S. aureus* strain Newman (*16*, *17*, *22*), which has been laboratory adapted over many decades (*43*), and may differ in virulence compared to recent clinical isolates. A9^−/−^ mice systemically infected with the methicillin-resistant *S. aureus* (MRSA) strain USA300 (LAC) showed reduced mortality (Fig. 1A), despite similar weight loss (fig. S1A). In addition, decreased bacterial burdens were observed specifically in the heart 4 days postinfection (dpi) compared to C57BL/6J wild-type (WT) mice (Fig. 1B). While these results were similar to what was obtained using Newman (*22*), they did not fully recapitulate every phenotype in the A9^−/−^ mice (*16*, *17*), as bacterial burdens in WT and A9^−/−^ mice were comparable in the kidney and liver (Fig. 1B). These discrepancies are likely strain specific, as differences in pathology between Newman and USA300 have been described during systemic infection (*44*). Despite these differences, A9^−/−^ mice are protected from USA300-mediated lethality and heart colonization by MRSA.

### S100A9 deficiency enhances suicidal NETosis

Neutrophils use multiple antimicrobial processes when engaging *S. aureus*. To test the impact of S100A9 on these processes, neutrophil function was assessed by flow cytometry (gating scheme provided in fig. S1B). Upon engaging *S. aureus*, neutrophils couple phagocytosis with the respiratory burst (*3*–*5*). Phagocytosis of fluorescently labeled *S. aureus* by WT and A9^−/−^ neutrophils was comparable (fig. S1C). Despite equal phagocytosis, the generation of ROS, as measured by dihydrorhodamine 123 (DHR123) staining, was increased in A9^−/−^ neutrophils relative to WT when stimulated with *S. aureus* (fig. S1D). However, this phenotype was not present in vivo as neutrophils isolated from the tissues of infected WT and A9^−/−^ mice produced comparable levels of ROS when stained with DHR123 ex vivo (fig. S1E). These results suggest that increased ROS production does not account for the protection of A9^−/−^ mice during infection.

**Fig. 2.**
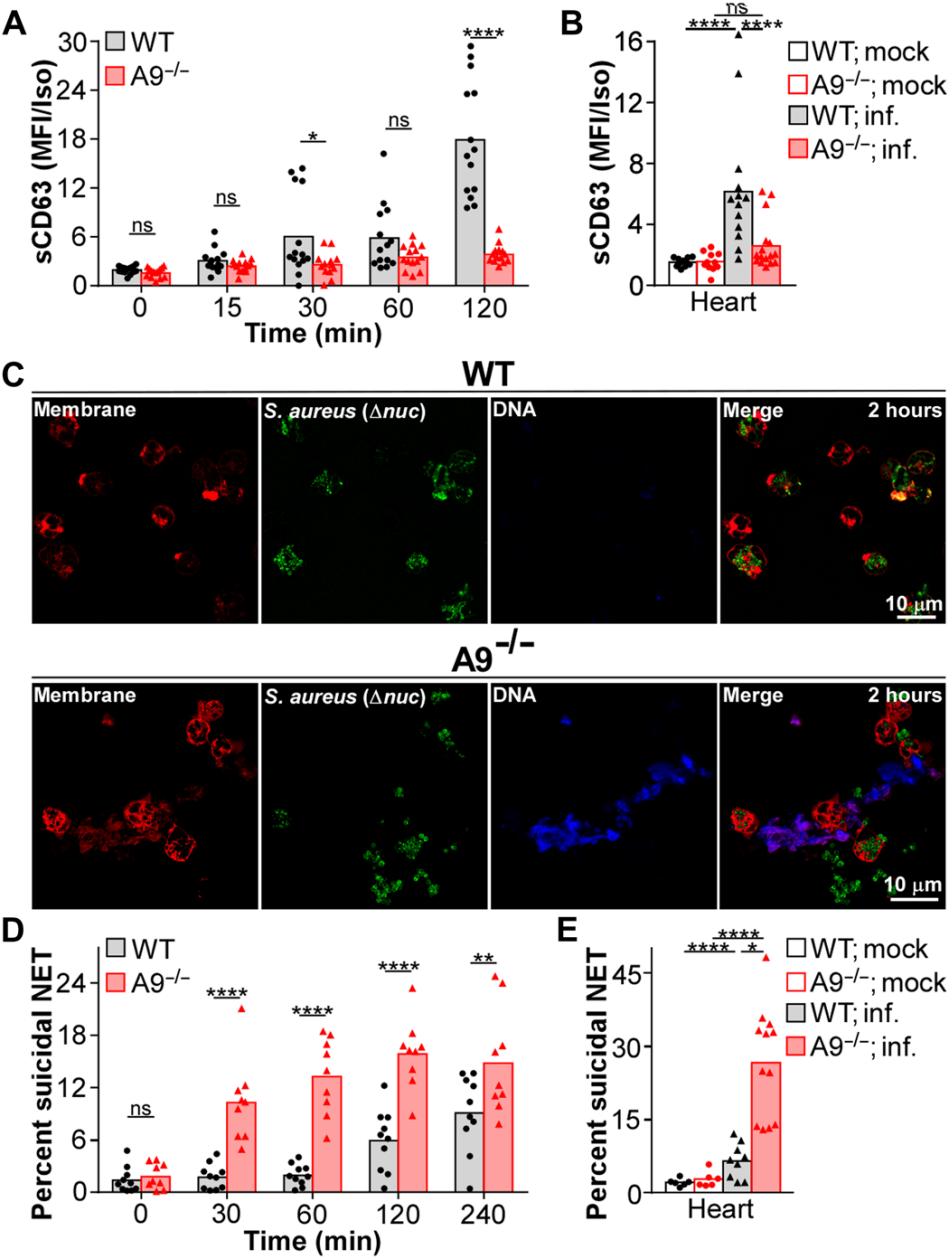
A9^−/−^ neutrophils have accelerated and more robust suicidal NETosis in response to *S. aureus*. (**A**) Neutrophils were cultured with *S. aureus* [multiplicity of infection (MOI) = 10] and primary degranulation [surface CD63 (sCD63)] by neutrophils (Ly6G^+^CD11b^+^) was quantified by flow cytometry. Median fluorescence intensity (MFI) normalized to an isotype (Iso) control. Each point represents neutrophils isolated from a single mouse (*n* = 14). (**B**) Mice were systemically infected (inf.) with *S. aureus* (CFU = 2 × 10^7^). At 4 dpi, organs were homogenized and primary degranulation by neutrophils was quantified by flow cytometry. MFI normalized to an isotype control. Each point represents a single mouse (mock, *n* = 11) (WT; inf., *n* = 13) (A9^−/−^; inf., *n* = 16). (**C** and **D**) Neutrophils were cultured with *S. aureus* (MOI = 10). (C) Representative images of neutrophils (red) stimulated for 2 hours with a nuclease-deficient (Δ*nuc*) strain of *S. aureus* (green) are provided. Extracellular DNA was stained using Helix NP Blue. (D) The percentage of neutrophils undergoing suicidal NETosis (dead: extracellular dsDNA^+^MPO^+^H3Cit^+^) in response to *S. aureus* was quantified by flow cytometry. Each point represents neutrophils isolated from a single mouse (*n* = 9). (**E**) Mice were systemically infected with *S. aureus* (CFU = 2 × 10^7^). At 4 dpi, organs were homogenized and the percentage of neutrophils undergoing suicidal NETosis was quantified by flow cytometry. Each point represents a single mouse (mock, *n* = 6) (WT; inf., *n* = 9) (A9^−/−^; inf., *n* = 12). Two-way ANOVA with (A and D) Sidak’s or (B and E) Tukey multiple comparisons test (*P ≤ 0.05, **P ≤ 0.01, and ****P ≤ 0.0001).

Neutrophils release granules containing AMPs in response to *S. aureus* (*6*, *7*). To quantify degranulation, neutrophils were stimulated with *S. aureus*, and the abundance of CD63 (primary degranulation) and CD35 (secretory degranulation) on the surface of the cell was quantified by flow cytometry. When cultured with *S. aureus*, primary degranulation was reduced in A9^−/−^ neutrophils compared to WT after 120 min (Fig. 2A and fig. S1F). Not all degranulation was restricted in A9^−/−^ neutrophils, as secretory degranulation was comparable to WT (fig. S1G). Furthermore, consistent with in vitro observations, primary degranulation by neutrophils was decreased in the heart and liver but comparable in the kidney of A9^−/−^ mice relative to WT mice, 4 dpi (Fig. 2B and fig. S1H). These results indicate that primary degranulation is impaired in A9^−/−^ neutrophils responding to *S. aureus* and that reduced primary degranulation in the heart coincides with reduced bacterial burdens (Fig. 1B).

Release of enzymes from primary granules into the cytosol is a precursor to NETosis (*45*, *46*); therefore, reduced primary degranulation could imply elevated NETosis. To identity NETs, a nonmembrane permeable dye was used to fluorescently stain the DNA backbone of NETs. After a 2-hour culture with *S. aureus*, extracellular DNA structures consistent with NET formation were more prevalent in cultures containing A9^−/−^ neutrophils compared to WT by confocal microscopy (Fig. 2C). To more accurately quantify vital and suicidal NETosis, we modified a flow cytometry–based assay that has been applied in vitro and in vivo as a high-throughput, quantitative method to identify neutrophils undergoing NETosis (*47*–*51*). Neutrophils undergoing NETosis were identified by the presence of extracellular double-stranded DNA (dsDNA), MPO, and histone citrulline (H3Cit), while a fluorescent dye allowed for monitoring of the cell membrane integrity to differentiate vital from suicidal NETosis. Thus, neutrophils with permeabilized cell membranes that are positive for extracellular dsDNA, MPO, and H3Cit were defined as having undergone suicidal NETosis, and neutrophils positive for extracellular dsDNA, MPO, and H3Cit and with intact cell membranes were defined as having undergone vital NETosis (gating scheme provided in fig. S2A). WT and A9^−/−^ neutrophils cultured with *S. aureus* demonstrated similar kinetics of vital NETosis, maximizing at 30 min (fig. S2B). However, the percentage of A9^−/−^ neutrophils undergoing suicidal NETosis was heightened from 30 to 240 min relative to WT (Fig. 2D and fig. S2C). Consistent with increased suicidal NETosis, supernatants from cultures containing A9^−/−^ neutrophils contained higher concentrations of NET DNA compared to supernatants isolated from cultures with WT neutrophils (fig. S2D). The accelerated suicidal NETosis by A9^−/−^ neutrophils occurs before nuclease secretion by *S. aureus*. Specifically, stimulating the Sae sensory system of *S. aureus* to promote nuclease expression (*52*, *53*) required 2 hours for nuclease activity in the supernatant to exceed the background activity observed in cultures containing a *S. aureus* strain lacking nuclease (Δ*nuc*; fig. S2E). These data indicate an accelerated and more robust suicidal NETosis by A9^−/−^ neutrophils in response to *S. aureus*.

To confirm that the flow cytometry–based strategy was monitoring NETosis and not other forms of cell death, an array of controls were used. First, the use of isotype control antibodies demonstrated that nonspecific antibody binding did not account for the positive staining of MPO and H3Cit (fig. S2F). Second, we used *Padi4^tm1.1Kmow^*/J (PAD4^−/−^) neutrophils that do not undergo NETosis in response to most stimuli (*54*). Consistent with an inability to NET, PAD4^−/−^ neutrophils have substantially reduced extracellular MPO and H3Cit compared to WT neutrophils in response to *S. aureus* (fig. S2F). In addition, neutrophils cultured with a strain of *S. aureus* with reduced lytic activity (*agr::tet*) (*55*) had comparable NETosis to a WT strain (fig. S2G). Last, to identify whether NET antigens produced by neutrophils undergoing NETosis can contaminate neighboring non-NETing neutrophils, a coculture of WT and fluorescently labeled PAD4^−/−^ neutrophils were stimulated with *S. aureus*. Only WT neutrophils robustly stained positive for NETosis (fig. S2H), indicating that accumulation of NET antigens on neutrophils not undergoing NETosis only marginally contributes to positive staining. These results demonstrate that the flow cytometry–based approach is specific to neutrophils undergoing NETosis and is not confounded by alternative forms of cell death.

Extracellular calprotectin acts as a chemoattractant (*18*, *19*) and DAMP (*20*, *21*), which may influence the neutrophil response to *S. aureus*. Therefore, neutrophils were cultured with *S. aureus* in the presence of recombinant calprotectin. The addition of exogenous calprotectin had no effect on degranulation or NETosis (fig. S3, A to C), indicating that the endogenous expression of S100A9 regulates neutrophil function in response to *S. aureus*.

To determine whether the heightened suicidal NETosis observed in vitro was present in A9^−/−^ mice, mice were infected and the percentage of neutrophils undergoing NETosis was quantified by flow cytometry 4 dpi. Coinciding with reduced primary degranulation (Fig. 2B) and bacterial burdens (Fig. 1B), suicidal NETosis was increased in the heart of A9^−/−^ mice but not significantly different in the liver and kidney relative to WT (Fig. 2E and fig. S3D). Vital NETosis was comparable between WT and A9^−/−^ mice (fig. S3E). An enzyme-linked immunosorbent assay (ELISA)–based approach to identifying NET structures has been used successfully to quantify NET DNA in vivo (*56*–*58*). Consistent with an increased percentage of neutrophils undergoing suicidal NETosis, an increased abundance of NETs was quantified from the supernatant of heart tissue from A9^−/−^ mice compared to WT by ELISA (fig. S3F). Thus, A9^−/−^ neutrophils in the heart have the strongest skewing toward suicidal NETosis during *S. aureus* infection coinciding with the largest decrease in bacterial burdens.

### Mitochondrial O_2_^−^ increases suicidal NETosis in the heart

**Fig. 3.**
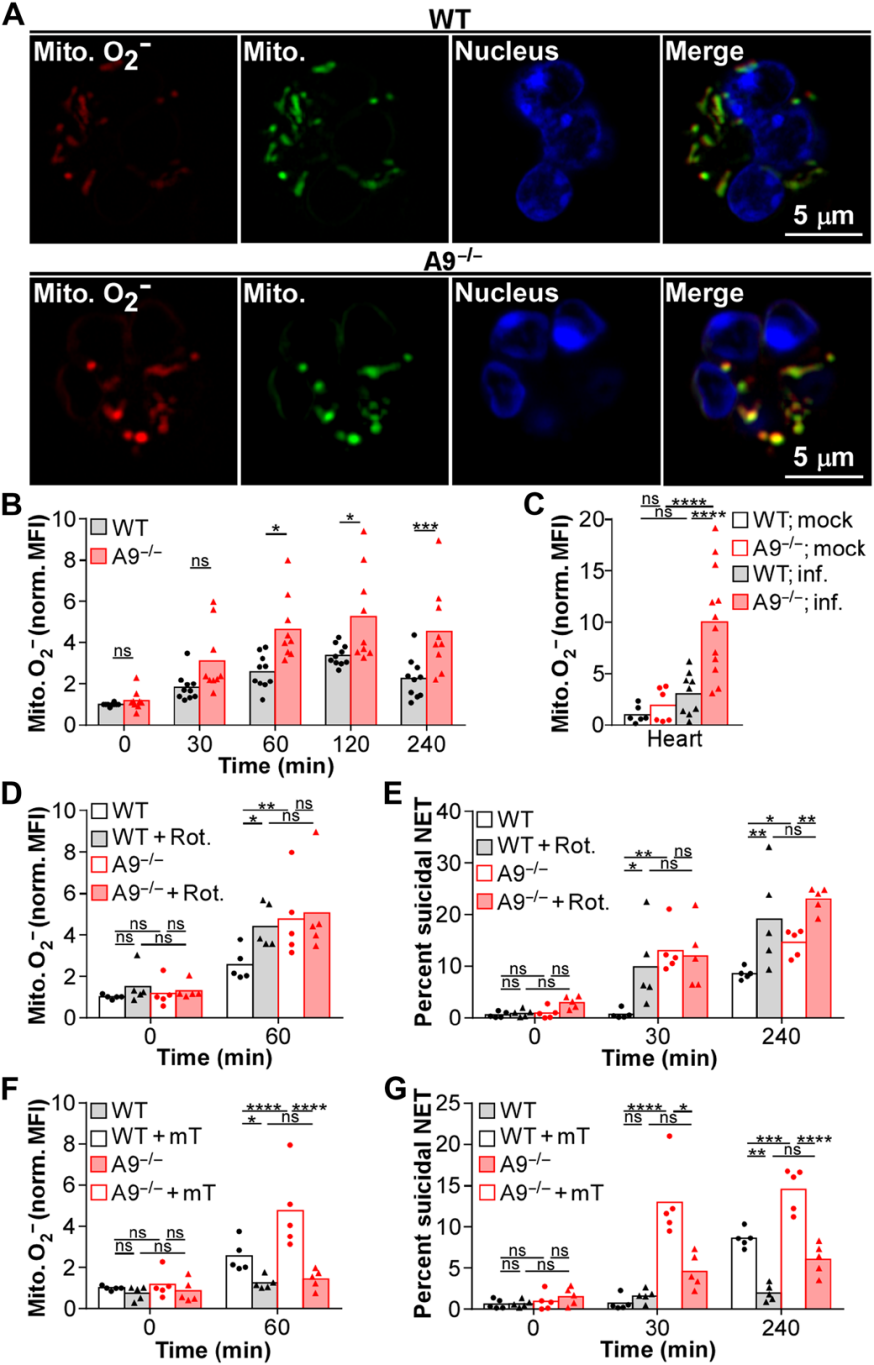
Increased mitochondrial O_2_^−^ heightens suicidal NETosis in A9^−/−^ neutrophils responding to *S. aureus*. (**A** and **B**) Neutrophils were cultured with *S. aureus* (MOI = 10). (A) Neutrophils were stimulated with *S. aureus* for 1 hour, and representative images of mitochondrial (Mito.) O_2_^−^ (red; MitoSOX) and biomass (green; MitoTracker) are provided. Nuclear DNA was stained (blue; Hoechst). (B) Neutrophils (Ly6G^+^CD11b^+^) were cultured with *S. aureus*, and production of mitochondrial O_2_^−^ was quantified by flow cytometry. MitoSOX MFI was normalized by MitoTracker MFI. Each point represents neutrophils isolated from a single mouse (*n* = 9). (**C**) Mice were systemically infected (inf.) with *S. aureus* (CFU = 2 × 10^7^). At 4 dpi, organs were homogenized and production of mitochondrial O_2_^−^ in neutrophils was quantified in the heart by flow cytometry. MitoSOX MFI was normalized by MitoTracker MFI. Each point represents a single mouse (mock, *n* = 6) (WT; inf., *n* = 9) (A9^−/−^; inf., *n* = 12). (**D** to **G**) Neutrophils pretreated with (D and E) rotenone (Rot.; 0.5 μM) for 15 min or (F and G) MitoTEMPO (mT; 0.5 μM) for 2 hours were cultured with *S. aureus* (MOI = 10). Neutrophils were stimulated with *S. aureus*, and (D and F) the production of mitochondrial O_2_^−^ and (E and G) the percentage of neutrophils undergoing suicidal NETosis (Dead: extracellular dsDNA^+^MPO^+^H3Cit^+^) were quantified by flow cytometry. (D and F) MitoSOX MFI was normalized by MitoTracker MFI. Each point represents neutrophils isolate from a single mouse (*n* = 5). Two-way ANOVA with (B) Sidak’s or (C to G) Tukey multiple comparisons test (*P ≤ 0.05, **P ≤ 0.01, ***P ≤ 0.001, and ****P ≤ 0.0001).

NADPH oxidase–derived ROS mediates suicidal NETosis (*59*); however, modulation of NADPH oxidase activity likely does not account for S100A9 regulation of suicidal NETosis since the respiratory burst is comparable between A9^−/−^ and WT neutrophils in vivo (fig. S1F). An NADPH oxidase–independent form of NETosis, relying solely on mitochondrial-derived ROS, has been described (*60*). Therefore, intracellular S100A9 could influence suicidal NETosis by altering the production of mitochondrial-derived ROS. To quantify mitochondrial O_2_^−^ generation, neutrophils were stained with a fluorescent dye that localizes to mitochondrial structures and fluoresces upon oxidation by O_2_^−^. Consistent with a role for mitochondrial O_2_^−^, A9^−/−^ neutrophils cultured with *S. aureus* maintained higher levels of mitochondrial O_2_^−^ from 60 to 240 min compared to WT (Fig. 3, A and B, and fig. S4A). Further, mitochondrial O_2_^−^ production by neutrophils was increased in the heart but comparable in the kidney and liver of A9^−/−^ mice relative to WT, 4 dpi (Fig. 3C and fig. S4B). Therefore, A9^−/−^ neutrophils in the heart produce more mitochondrial O_2_^−^ in response to *S. aureus*, which correlates with increased suicidal NETosis.

To determine whether increased mitochondrial O_2_^−^ alters the response of A9^−/−^ neutrophils to *S. aureus*, neutrophils were treated with rotenone to increase or MitoTEMPO to quench mitochondrial O_2_^−^ production. In addition to increasing mitochondrial O_2_^−^ levels (Fig. 3D and fig. S4C), rotenone accelerated and enhanced suicidal NETosis (Fig. 3E and fig. S4D) and decreased primary degranulation (fig. S4E) in response to *S. aureus*, to levels similar to A9^−/−^. Conversely, quenching mitochondrial O_2_^−^ using MitoTEMPO (Fig. 3F and fig. S4C) reduced the percentage of A9^−/−^ neutrophils undergoing suicidal NETosis in response to *S. aureus* (Fig. 3G and fig. S4D), while primary degranulation was modestly increased (fig. S4E). Suicidal NETosis was fully impaired in MitoTEMPO-treated WT neutrophils cultured with *S. aureus* (Fig. 3G). Mitochondrial O_2_^−^ did not influence all forms of NETosis as vital NETosis was unaffected by rotenone and MitoTEMPO treatments (fig. S4F). Thus, mitochondrial O_2_^−^ regulates suicidal NETosis in response to *S. aureus*.

**Fig. 4.**
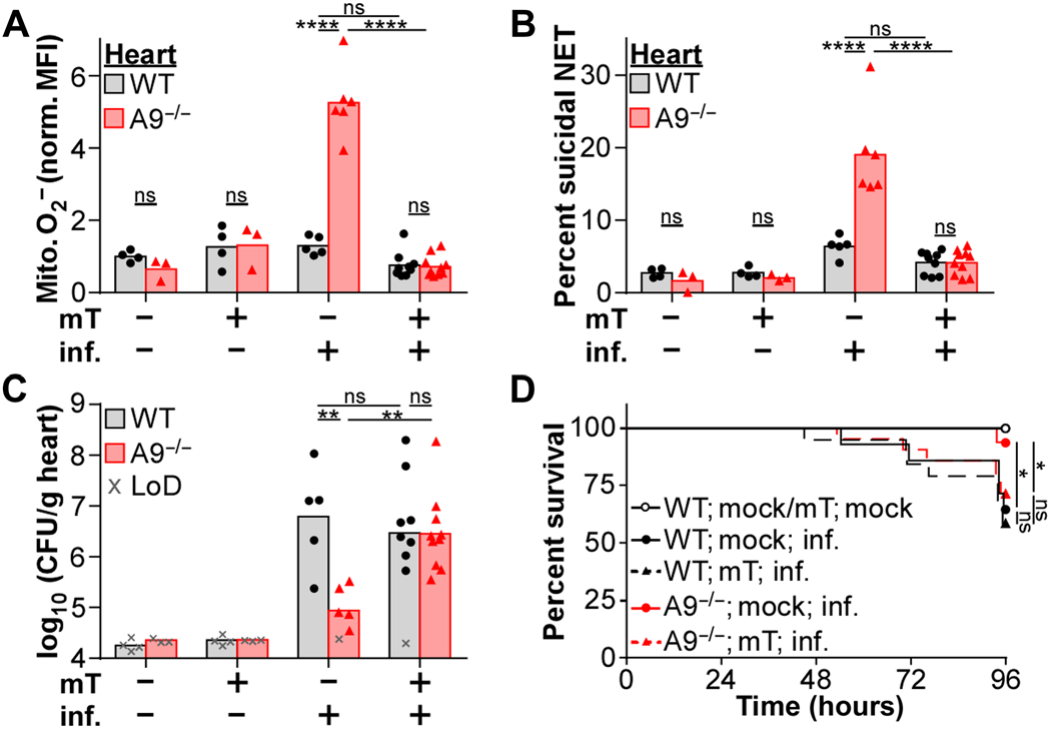
MitoTEMPO treatment of A9^−/−^ mice reduces suicidal NETosis coinciding with enhanced lethality and heart colonization. Mice were treated with MitoTEMPO (mT; 0.7 mg/kg) by intraperitoneal injection 24 hours before and every 24 hours during systemic infection (inf.) with *S. aureus* (A to C, CFU = 2 × 10^7^; D, CFU = 5 × 10^7^). (**A** to **C**) At 4 dpi, organs were homogenized, and (A) the production of mitochondrial O_2_^−^ by neutrophils (Ly6G^+^CD11b^+^) and (B) the percentage of neutrophils undergoing suicidal NETosis (Dead: extracellular dsDNA^+^MPO^+^H3Cit^+^) were quantified by flow cytometry, and (C) CFU was counted using spot plating (LoD). (A) MitoSOX MFI was normalized by MitoTracker MFI. Each point represents a single mouse (mock; mock/mT; WT, *n* = 4) (mock; mock/mT; A9^−/−^, *n* = 3) (inf.; mock; WT, *n* = 5) (inf.; mock; A9^−/−^, *n* = 6) (inf.; mT; WT, *n* = 9) (inf.; mT; A9^−/−^, *n* = 10). (**D**) During the infection, mouse survival was monitored. Each point represents the percentage of living mice (mock; mock; WT/A9^−/−^, *n* = 7) (mock; mT; WT/A9^−/−^, *n* = 8) (inf.; mock; WT, *n* = 13) (inf.; mock; A9^−/−^, *n* = 15) (inf.; mT; WT, *n* = 19) (inf.; mT; A9^−/−^, *n* = 21). (A to C) Two-way ANOVA with Tukey multiple comparisons test or (D) log-rank (Mantel-Cox) test (*P ≤ 0.05, **P ≤ 0.01, and ****P ≤ 0.0001).

Since MitoTEMPO treatment reduces suicidal NETosis in vitro, treating A9^−/−^ mice with MitoTEMPO to reduce the production of mitochondrial O_2_^−^ was hypothesized to enhance disease pathology in the heart during *S. aureus* infection. Therefore, mice were intraperitoneally injected with MitoTEMPO daily starting 24 hours before infection. Treatment with MitoTEMPO during infection was sufficient to decrease mitochondrial O_2_^−^ levels in neutrophils within the heart, liver, and kidney of A9^−/−^ mice to levels comparable to WT (Fig. 4A and fig. S5A). The activity of MitoTEMPO was specific to mitochondrial O_2_^−^ as overall ROS levels in neutrophils were unchanged (fig. S5B). Furthermore, while vital NETosis was unaffected by MitoTEMPO (fig. S5C), the percentage of neutrophils undergoing suicidal NETosis was reduced (Fig. 4B and fig. S5D), and primary degranulation was increased (fig. S5E) to similar levels as WT in the heart of A9^−/−^ mice. Corresponding with reduced suicidal NETosis, MitoTEMPO treatment increased bacterial burdens in the heart (Fig. 4C) and mortality (Fig. 4D) of A9^−/−^ mice to levels comparable to WT. Treatment with MitoTEMPO did not alter mouse weight (fig. S5F), NETosis (fig. S5, C and D), or bacterial burdens (fig. S5G) in the liver and kidney. These results demonstrate that increased mitochondrial O_2_^−^ benefits neutrophil function in the heart by enhancing suicidal NETosis in response to *S. aureus*, thereby reducing bacterial burdens.

### Modulating NET formation alters *S. aureus* disease outcome

**Fig. 5.**
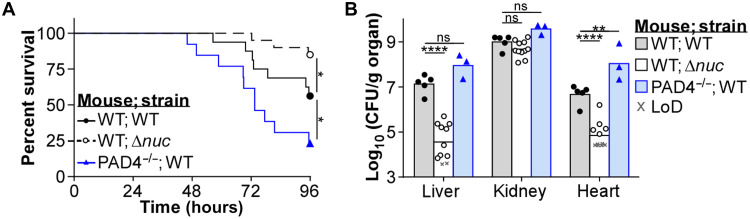
NET formation contributes to the protection of mice during systemic infection with *S. aureus*. Mice were systemically infected with *S. aureus* (CFU = 2 × 10^7^). (**A**) During the infection, mouse survival was monitored. Each point represents the percentage of living mice (WT; WT, *n* = 16) (WT; Δ*nuc*, *n* = 20) (PAD4^−/−^; WT, *n* = 13). (**B**) At 4 dpi, organs were homogenized and CFU was counted using spot plating (LoD). Each point represents a single mouse (WT; WT, *n* = 5) (WT; Δ*nuc*, *n* = 11) (PAD4^−/−^; WT, *n* = 3). (A) Log-rank (Mantel-Cox) test or (B) two-way ANOVA with Tukey multiple comparisons test (*P ≤ 0.05, **P ≤ 0.01, and ****P ≤ 0.0001).

While increased suicidal NETosis correlates to reduced heart colonization during systemic infection (Figs. 2 and 4), the antibacterial activity of NETs can vary in response to *S. aureus* (*10*–*12*). In addition, the role of NET formation in the heart during *S. aureus* infection has not been explored. If suicidal NETosis is protective during systemic *S. aureus* infections, then PAD4^−/−^ mice, where the neutrophils cannot undergo NETosis in response to most stimulations (fig. S2F) (*54*), should exhibit increased susceptibility. Following infection, PAD4^−/−^ mice had increased mortality relative to WT (Fig. 5A) despite decreased weight loss (fig. S6A). Furthermore, of mice surviving to 4 dpi, bacterial burdens were increased in the heart of PAD4^−/−^ mice compared to WT mice (Fig. 5B). The secretion of a nuclease allows *S. aureus* to escape NET-mediated antibacterial activity by degrading the DNA backbone of NETs (*10*). Consistent with NET formation protecting the host, mice systemically infected with Δ*nuc* showed reduced mortality (Fig. 5A) and weight loss (fig. S6A) relative to WT infected mice. Further, lower bacterial burdens were observed in the heart and liver of mice infected with a Δ*nuc* strain relative to WT 4 dpi (Fig. 5B). These results demonstrate that NET formation contributes to protecting the host from systemic *S. aureus* infections and reducing bacterial burdens in the heart.

### NET formation by A9^−/−^ neutrophils enhances *S. aureus* killing in the presence of Mφs

**Fig. 6.**
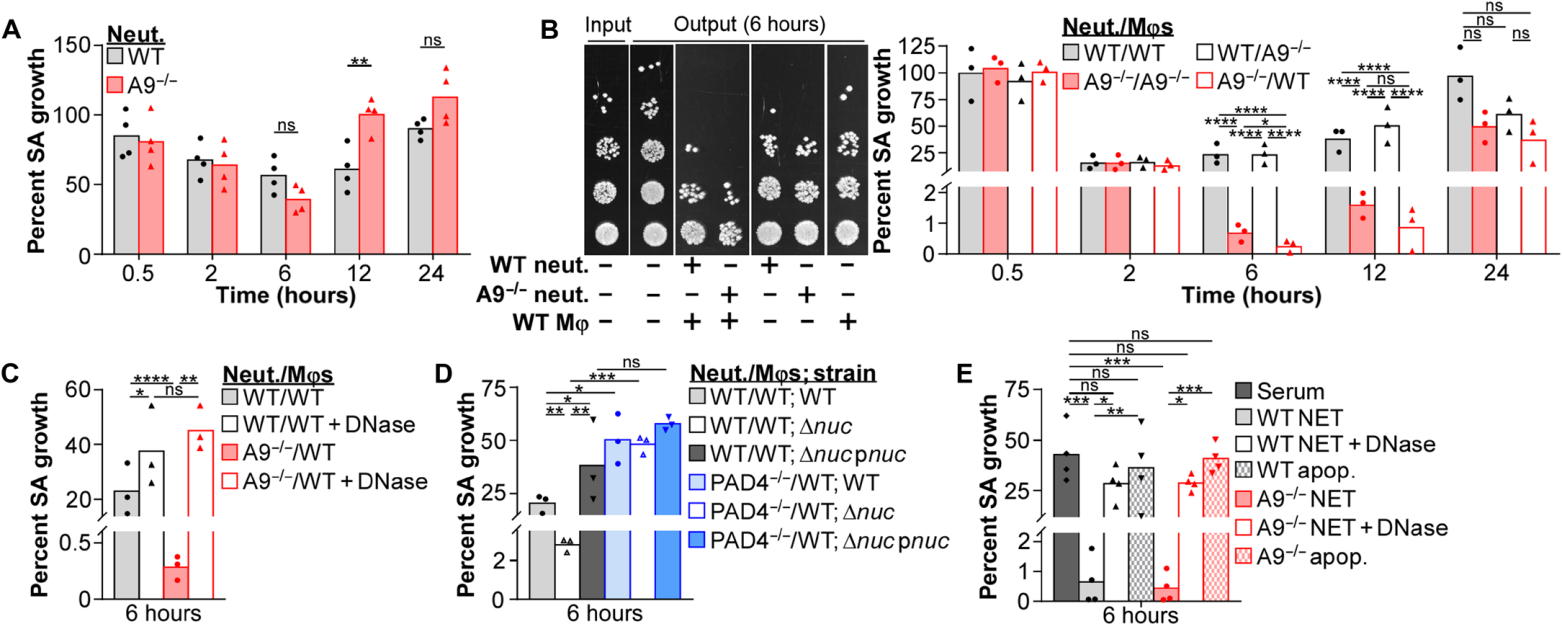
NET formation from A9^−/−^ neutrophils better restrict *S. aureus* growth in the presence of Mφs. Immune cells were cultured with *S. aureus* (MOI = 1). (**A**) Neutrophil (Neut.) restriction of *S. aureus* growth was quantified by CFU spot plating. Percent growth of *S. aureus* (SA) calculated relative to *S. aureus* growth in the absence of neutrophils. Each point represents the mean result (biological triplicate) of neutrophils isolated from a single mouse (*n* = 4). (**B** to **D**) Neutrophil-Mφ coculture (ratio = 1:1) restriction of *S. aureus* growth was quantified by CFU spot plating. Percent growth of *S. aureus* calculated relative to *S. aureus* growth in the absence of immune cells. (B) Representative spot plating from a single experiment is provided. (C) Immune cells cultured with *S. aureus* in the presence of deoxyribonuclease (DNase) (8 U/ml). Each point represents the mean result (biological triplicate) of immune cells isolated from a single mouse (*n* = 3). (**E**) Mφ restriction of *S. aureus* growth was quantified by CFU spot plating. NETs or apoptotic debris (apop.) used for *S. aureus* opsonization were isolated from neutrophils stimulated with phorbol 12-myristate 13-acetate (PMA) (100 nM) for 4 hours or an anti-FAS antibody (100 ng/ml) for 16 hours. Percent growth of *S. aureus* calculated relative to *S. aureus* growth in the absence of immune cells. Mφs cultured with *S. aureus* in the presence of DNase (8 U/ml). Each point represents the mean result (biological triplicate) of Mφs isolated from a single mouse (*n* = 4). Two-way ANOVA with (A) Sidak’s or (B to E) Tukey multiple comparisons test (*P ≤ 0.05, **P ≤ 0.01, ***P ≤ 0.001, and ****P ≤ 0.0001).

NET formation aids in protecting the host from systemic *S. aureus* infections (Fig. 5). The accelerated and robust suicidal NETosis by A9^−/−^ neutrophils in response to *S. aureus* (Fig. 2) provides a powerful tool to assess NET-related biology. Therefore, we hypothesized that enhanced suicidal NETosis by A9^−/−^ neutrophils would render them more bactericidal. However, in vitro, A9^−/−^ neutrophils did not restrict *S. aureus* growth better than WT (Fig. 6A). Thus, suicidal NETosis alone does not account for the increased protection of A9^−/−^ mice from systemic *S. aureus* infections.

Neutrophils and Mφs can cooperatively combat intracellular bacterial pathogens during infection (*33*–*36*), but whether similar cooperative mechanisms exist in combating extracellular pathogens, such as *S. aureus*, is unclear. During *S. aureus* infection, the ratio of neutrophils to Mφs is comparable in the liver and kidney of WT and A9^−/−^ mice as neutrophils outnumbered Mφs by 2-fold in the liver and 6.5-fold in the kidney (fig. S6B). In the heart, the neutrophil/Mφ ratio was 4.6 in WT mice compared to 1.3 in A9^−/−^ mice, a difference that can be attributed to a reduction of live neutrophils observed in the heart of A9^−/−^ mice (fig. S6B) (*22*) and possibly due to increased suicidal NETosis (Fig. 2E). Cocultures containing neutrophils isolated from the bone marrow and bone marrow–derived Mφs at a ratio of 1:1 were stimulated with *S. aureus*. Consistent with increased antibacterial activity, cocultures containing neutrophils and Mφs restricted *S. aureus* growth better (Fig. 6B) than cultures containing equal numbers of neutrophils (Fig. 6A) or Mφs (fig. S6C) alone. Further, cocultures containing A9^−/−^ neutrophils with either WT or A9^−/−^ Mφs restricted *S. aureus* growth better (≥1.3-log) than cocultures containing WT neutrophils at 6 and 12 hours (Fig. 6B). Skewing the coculture so that either Mφs outnumbered neutrophils or neutrophils outnumber Mφs at a ratio of 5:1 or higher rapidly ablated the enhanced bactericidal activity of cocultures containing A9^−/−^ neutrophils (fig. S6D). These results demonstrate that A9^−/−^ neutrophils have increased antibacterial activity in the presence of Mφs.

To determine whether enhanced suicidal NETosis accounts for increased bacterial killing in the presence of Mφs, neutrophil-Mφ cocultures were treated with deoxyribonuclease (DNase) to degrade the DNA backbone of NETs, as previously described (*11*, *56*). DNase treatment ablated the improved restriction of *S. aureus* growth in cocultures containing A9^−/−^ neutrophils to levels lower than cocultures containing WT neutrophils (Fig. 6C). A similar, but less pronounced, phenotype was observed in cultures containing only neutrophils (fig. S6E), but no effect was observed on Mφ killing of *S. aureus* (fig. S6F). In addition, neutrophil-Mφ cocultures containing PAD4^−/−^ neutrophils failed to restrict *S. aureus* growth and cocultures containing WT neutrophils (Fig. 6D). Thus, NET formation provides an advantage for neutrophils engaging *S. aureus*, and this is enhanced by the presence of Mφs.

Nuclease secretion protects *S. aureus* from NET-mediated killing (*10*). Since NET formation in the presence of Mφs contributes to bacterial killing, we hypothesized that growth of Δ*nuc* should be restricted in neutrophil-Mφ cocultures. In neutrophil-Mφ cocultures, Δ*nuc* growth was reduced relative to WT *S. aureus* (Fig. 6D). Expression of nuclease in trans (Δ*nuc* p*nuc*) complemented this phenotype to levels comparable to cocultures containing WT *S. aureus*. A similar, but less pronounced, phenotype was observed in cultures containing only neutrophils (fig. S6G), but the growth of WT and Δ*nuc* strains were comparably restricted in cultures containing Mφs alone (fig. S6H). Combined with the observation that detectable nuclease activity in the supernatant occurs after 2 hours (fig. S2E), these results suggest that the accelerated and more robust suicidal NETosis by A9^−/−^ neutrophils may outpace and/or overwhelm nuclease activity, giving A9^−/−^ neutrophils an advantage when combating *S. aureus* in the presence of Mφs.

NET formation in the presence of Mφs limits *S. aureus* growth (Fig. 6, B to D), but neutrophils may perform other functions contributing to *S. aureus* killing. To determine whether NET formation is sufficient to increase Mφ-mediated killing of *S. aureus*, NET structures were isolated from the supernatant of phorbol 12-myristate 13-acetate (PMA)–stimulated neutrophils as previously described (*61*). Equal concentrations of NETs from WT or A9^−/−^ neutrophils were added to *S. aureus* in culture media and then transferred to Mφs. NETs isolated from WT and A9^−/−^ neutrophils comparably enhanced Mφ restriction of *S. aureus* growth (Fig. 6E), demonstrating that NETs from A9^−/−^ neutrophils are not more antibacterial than WT. Further, DNase treatment reduced the restriction of *S. aureus* growth by Mφs to levels comparable to using serum as an opsonin. Stimulation with an anti-Fas antibody to trigger apoptosis caused neutrophils to release less DNA into the supernatant (fig. S6I) and failed to enhance Mφ killing of *S. aureus* (Fig. 6E), suggesting that not all forms of neutrophil cell death enhance the antibacterial activity of Mφs. These data demonstrate that NETs increase the antibacterial activity of Mφs toward *S. aureus*.

### NETs enhance phagocytosis of *S. aureus* by Mφs

**Fig. 7.**
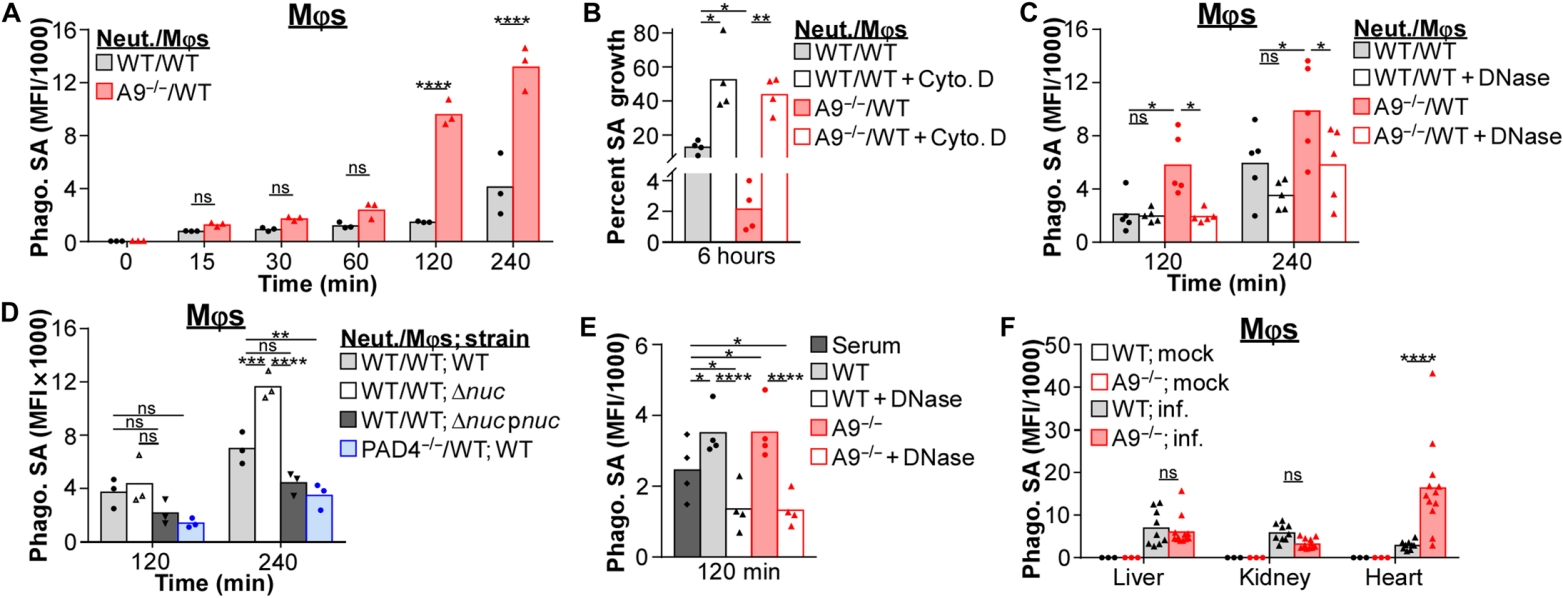
NET formation enhances phagocytosis of *S. aureus* by Mφs. (**A** to **E**) Immune cells were cultured with fluorescently labeled *S. aureus* (MOI = 10). (A) Phagocytosis of *S. aureus* by Mφs in coculture (ratio = 1:1) with neutrophils (Neut.) was quantified by flow cytometry. Background MFI was subtracted from each time point. Each point represents immune cells isolated from a single mouse (*n* = 3). (B) Mφs were pretreated with cytochalasin D (Cyto. D; 10 μg/ml) for 1 hour before adding neutrophils and *S. aureus*. Percent growth of *S. aureus* was calculated relative to *S. aureus* growth in the absence of immune cells. Each point represents the mean result (biological triplicate) of immune cells isolated from a single mouse (*n* = 4). (C and D) Phagocytosis of *S. aureus* by Mφs in coculture with neutrophils was quantified by flow cytometry. Background MFI was subtracted from each time point. (C) Immune cells cultured with *S. aureus* in the presence of DNase (8 U/ml). Each point represents immune cells isolated from a single mouse (C, *n* = 5; D, *n* = 3). (E) Phagocytosis of *S. aureus* by Mφs was quantified by flow cytometry. Background MFI was subtracted from each time point. NETs used for *S. aureus* opsonization were isolated from neutrophils stimulated with PMA (100 nM) for 4 hours and cultured with Mφs in the presence of DNase (8 U/ml). Each point represents immune cells isolated from a single mouse (*n* = 4). (**F**) Mice were systemically infected with a fluorescent strain of *S. aureus* (p*SarA_sfGFP*; CFU = 2 × 10^7^). At 4 dpi, organs were homogenized and *S. aureus* levels within Mφs (CD11b^+^F4/80^+^Ly6G^−^) in the heart were quantified by flow cytometry. Background MFI from uninfected mice was subtracted from infected. Each point represents a single mouse (mock, *n* = 3) (WT; inf., *n* = 9) (A9^−/−^; inf., *n* = 12). Two-way ANOVA with (A) Sidak’s or (B to F) Tukey multiple comparisons test (*P ≤ 0.05, **P ≤ 0.01, ***P ≤ 0.001, and ****P ≤ 0.0001).

*S. aureus* has multiple strategies to avoid phagocytosis (*62*–*64*); however, entrapment of *S. aureus* in NETs may aid phagocytosis by Mφs. To quantify phagocytosis, immune cells were cultured with fluorescently labeled *S. aureus*. Phagocytosis of *S. aureus* by Mφs was enhanced in cocultures containing A9^−/−^ neutrophils relative to cocultures with WT neutrophils (Fig. 7A and fig. S7A). Phagocytosis by neutrophils in cocultures was comparable (fig. S7B). Pretreating Mφs with cytochalasin D prevented phagocytosis of *S. aureus* by Mφs in the cocultures but not neutrophils (fig. S7C) and prevented cocultures containing A9^−/−^ neutrophils from having enhanced antibacterial activity (Fig. 7B). These data suggest that phagocytosis by Mφs is required for A9^−/−^ neutrophils to enhance antibacterial activity in response to *S. aureus*.

To identify whether the enhanced phagocytosis by Mφs cocultured with A9^−/−^ neutrophils was NET dependent, cocultures were treated with DNase to degrade the DNA backbone of NETs. Treating cocultures containing A9^−/−^ neutrophils with DNase reduced phagocytosis of *S. aureus* by Mφs to levels similar to cocultures containing WT neutrophils (Fig. 7C). Consistent with NETs facilitating phagocytosis by Mφs, neutrophil-Mφ cocultures containing PAD4^−/−^ neutrophils did not increase phagocytosis of *S. aureus* by Mφs, while cocultures stimulated with Δ*nuc* enhanced phagocytosis of *S. aureus* by Mφs (Fig. 7D and fig. S7A). Last, adding isolated NETs from PMA-stimulated neutrophils to *S. aureus* increased phagocytosis by Mφs to greater levels than opsonizing *S. aureus* in serum, which was prevented by treating with DNase (Fig. 7E). Thus, NETs may act as a pseudo-opsonin that facilitates phagocytosis by Mφs.

To determine whether enhanced suicidal NETosis increases phagocytosis of *S. aureus* by Mφs in vivo, mice were infected with a fluorescent *S. aureus* strain (p*SarA_sfGFP*) (*65*). Consistent with increased suicidal NETosis (Fig. 2E), Mφs in the heart of A9^−/−^ mice had higher levels of internalized *S. aureus* relative to WT (Fig. 7F and fig. S7D), which was not observed in the kidney and liver (fig. S7D). Internalized *S. aureus* levels in neutrophils was decreased in the heart and kidney and comparable in the liver of A9^−/−^ mice (fig. S7E). These results demonstrate that increased suicidal NETosis correlates to enhanced internalization of *S. aureus* by Mφs, which may contribute to the specific reduction in bacterial burdens in the heart of A9^−/−^ mice.

### NETs transfer AMPs to Mφs during *S. aureus* infection

**Fig. 8.**
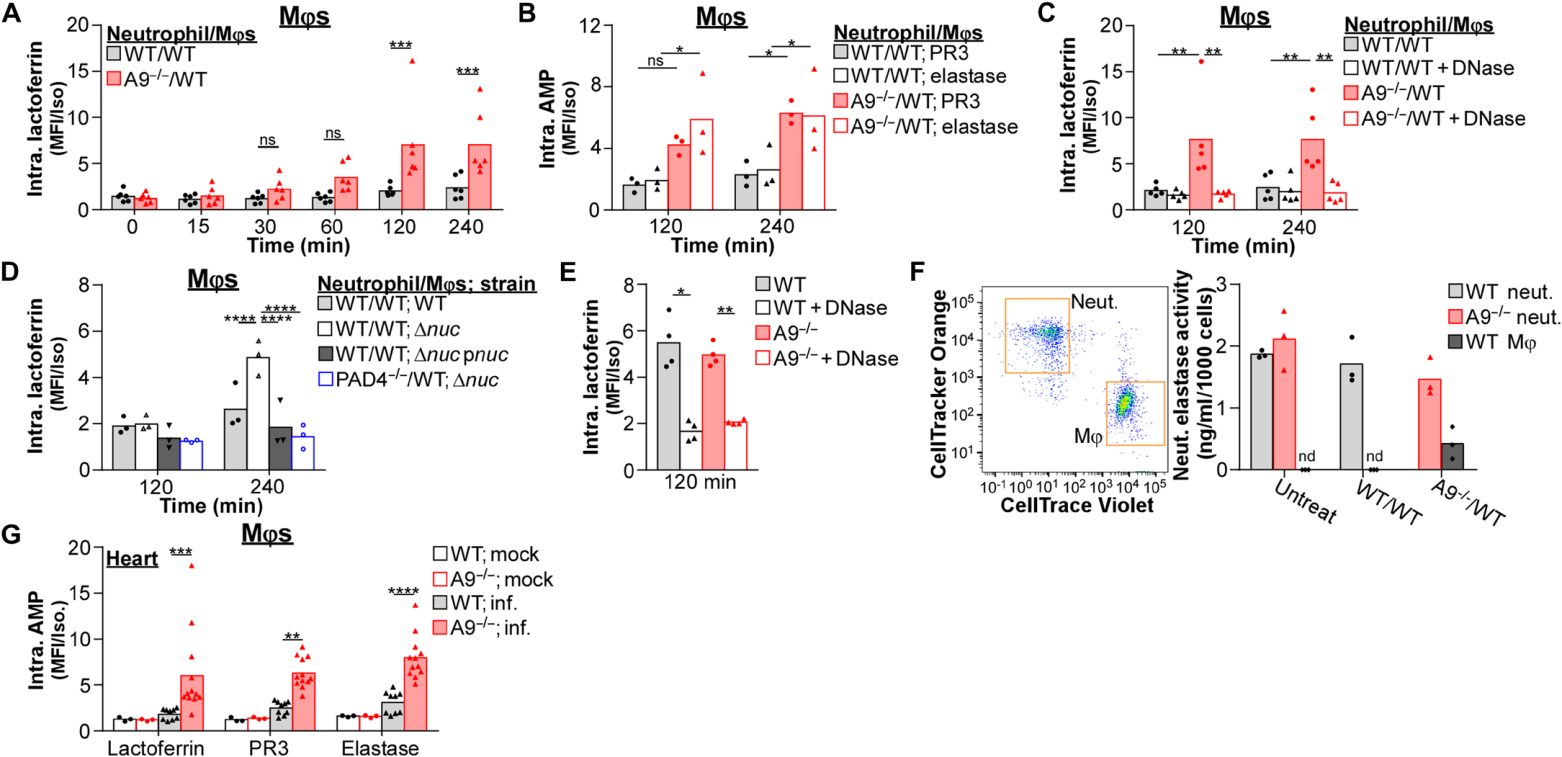
NET formation transfers neutrophil-specific AMPs to Mφs. (**A** to **F**) Immune cells were cultured with *S. aureus* (MOI = 10). (A to D) The intracellular (Intra.) abundance of lactoferrin, PR3, and elastase within Mφs in coculture (ratio = 1) with neutrophils (Neut.) following stimulation with *S. aureus* were quantified by flow cytometry. MFI was normalized to an isotype control. (C) Immune cells were cultured with *S. aureus* in the presence of DNase (8 U/ml). Each point represents immune cells isolated from a single mouse (A, *n* = 6; B and D, *n* = 3; C, *n* = 5). (E) Intracellular abundance of lactoferrin within Mφs following stimulation with *S. aureus* was quantified by flow cytometry. MFI was normalized to an isotype control. NETs used for *S. aureus* opsonization were isolated from neutrophils stimulated with PMA (100 nM) for 4 hours and cultured with Mφs in the presence of DNase (8 U/ml). Each point represents immune cells isolated from a single mouse (*n* = 4). (F) Cocultures were stimulated with *S. aureus*, and after 2 hours, neutrophils and Mφs were isolated by fluorescent sorting. Lysates from isolated cells were quantified for neutrophil elastase activity and normalized by cell number (nd, no activity detected). Each point represents the mean result (biological duplicate) of immune cells isolated from a single mouse (*n* = 3). (**G**) Mice were systemically infected with *S. aureus* (CFU = 2 × 10^7^). At 4 dpi, organs were homogenized and intracellular lactoferrin, PR3, and elastase levels within Mφs (CD11b^+^F4/80^+^Ly6G^−^) in the heart were quantified by flow cytometry. MFI was normalized by an isotype control. Each point represents a single mouse (mock, *n* = 3) (WT; inf., *n* = 9) (A9^−/−^; inf., *n* = 12). Two-way ANOVA with (A) Sidak’s or (B to E and G) Tukey multiple comparisons test (*P ≤ 0.05, **P ≤ 0.01, ***P ≤ 0.001, and ****P ≤ 0.0001).

Despite the antimicrobial activity of phagolysosomes, *S. aureus* can persist and replicate within Mφs (*23*, *31*, *32*). While engulfment of apoptotic neutrophils transfers AMPs to Mφs in response to intracellular bacterial pathogens (*33*–*36*), NET formation may provide an alternative means to transfer neutrophil-specific AMPs to Mφs in response to extracellular pathogens, such as *S. aureus*. Mφs expressed low levels of lactoferrin relative to neutrophils before stimulation (fig. S8A). However, Mφs in cocultures containing A9^−/−^ neutrophils accumulated higher levels of lactoferrin in response to *S. aureus* compared to cocultures with WT neutrophils after 120 min (Fig. 8A and fig. S8B). This phenotype was not unique to lactoferrin as the abundance of other neutrophil-specific AMPs, including proteinase 3 (PR3) and neutrophil elastase, was increased in Mφs from cocultures containing A9^−/−^ neutrophils relative to WT in response to *S. aureus* (Fig. 8B and fig. S8C). *S. aureus* did not cause AMPs to accumulate in Mφs in the absence of neutrophils (fig. S8D). DNase treatment of cocultures containing A9^−/−^ neutrophils reduced the abundance of AMPs in Mφs to levels comparable to cocultures with WT neutrophils (Fig. 8C and fig. S8E). Thus, NET formation transfers neutrophil-specific AMPs to Mφs coinciding with enhanced antibacterial activity of Mφs.

Since NET formation transfers AMPs to Mφs, we predicted that the Δ*nuc* strain of *S. aureus* would allow increased transfer compared to WT. Mφs in cocultures accumulated higher levels of AMPs in response to Δ*nuc* relative to cocultures stimulated with a WT strain (Fig. 8D and fig. S8, B, C, and F). Expression of nuclease in trans complemented the abundance of AMPs in Mφs to levels comparable to cocultures stimulated with a WT strain. In addition, Mφs in cocultures containing PAD4^−/−^ neutrophils have a decreased abundance of AMPs in response to Δ*nuc*, which are similar to levels observed using Δ*nuc* p*nuc*. Last, isolated NETs from PMA-stimulated neutrophils added to *S. aureus* were sufficient to increase the abundance of lactoferrin in Mφs, while DNase treatment prevented this accumulation (Fig. 8E). Therefore, NET formation acts as a conduit to transfer neutrophil-specific AMPs to Mφs, and secretion of nuclease by *S. aureus* combats AMP transfer.

It has been previously demonstrated that NETs transfer neutrophil elastase to Mφs and that elastase retains its activity within the Mφ (*56*). The elevated suicidal NETosis by A9^−/−^ neutrophils efficiently transfers AMPs to Mφs in response to *S. aureus* (Fig. 8, A to E, and fig. S8, B, C, E, and F). To determine whether neutrophil elastase transferred by A9^−/−^ neutrophils to Mφs retained enzymatic activity, cocultures were stimulated with *S. aureus*, and neutrophils and Mφs were fluorescently sorted after 120 min. Lysates from isolated immune cells were quantified for neutrophil elastase activity. Neutrophil elastase activity was not detected in Mφs from cocultures with WT neutrophils; however, in cocultures containing A9^−/−^ neutrophils, neutrophil elastase activity in Mφs was detectable (Fig. 8F), coinciding with the transfer of neutrophil elastase to Mφs (Fig. 8B). These results suggest that the enhanced suicidal NETosis by A9^−/−^ neutrophil transfers biologically active AMPs to Mφs in response to *S. aureus*.

To determine whether increased NET formation facilitates the accumulation of neutrophil-specific AMPs in Mφs in vivo, mice were infected, and lactoferrin, PR3, and elastase abundance in Mφs was quantified by flow cytometry. Correlating with increased suicidal NETosis (Fig. 2E), Mφs in the heart of A9^−/−^ mice accumulated higher levels of neutrophil-specific AMPs compared to WT mice (Fig. 8G and fig. S8G), which was not observed in the liver and kidney (fig. S8G). Neutrophil AMP levels were comparable across organs (fig. S8H). These results demonstrate that increased suicidal NETosis enhances transfer of neutrophil-specific AMPs to Mφs, which correlates with the specific reduction in bacterial burdens in the heart of A9^−/−^ mice.

### NET formation in the presence of Mφs enhances killing of multiple bacterial pathogens

**Fig. 9.**
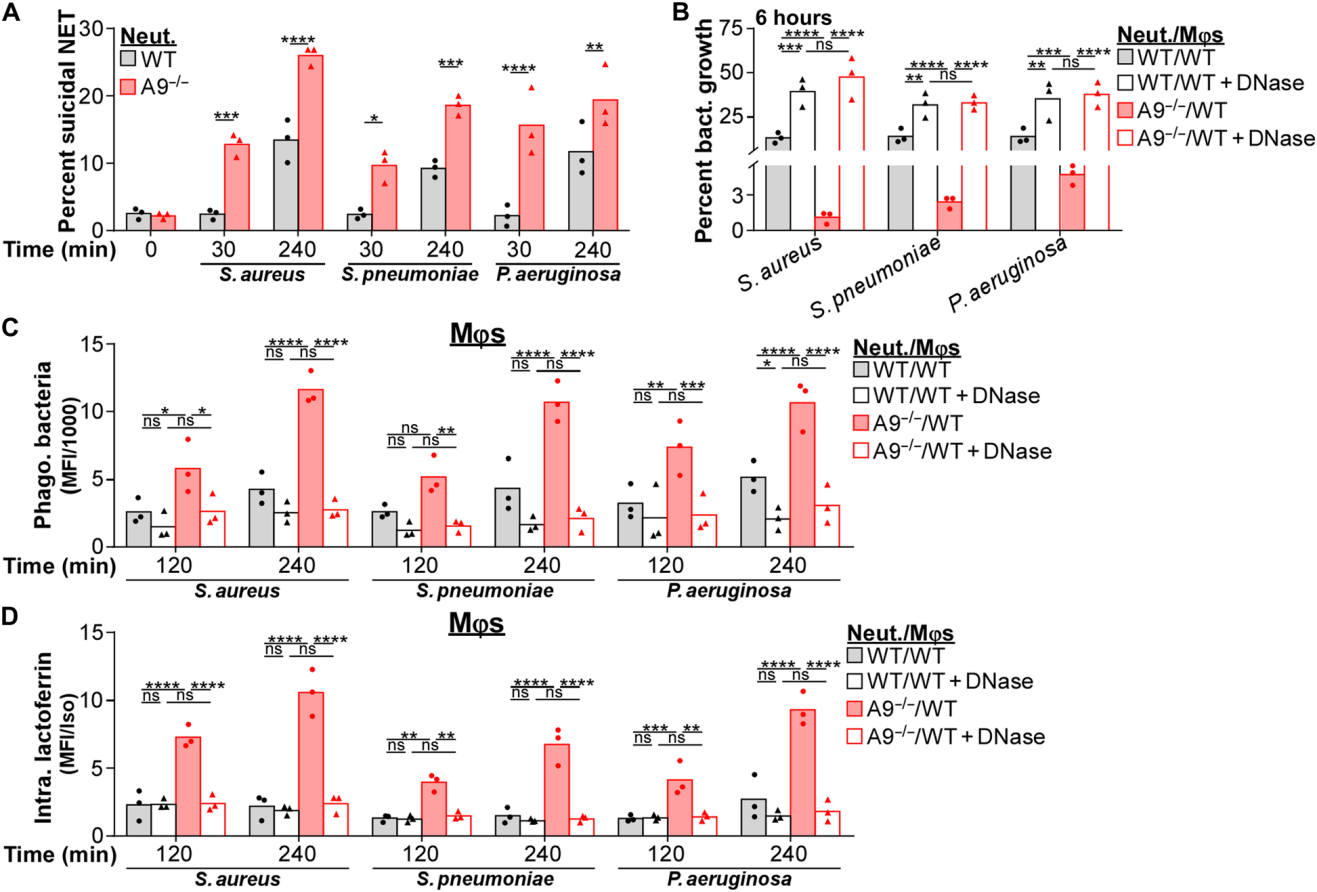
NET formation better restricts growth of multiple bacterial pathogens in the presence of Mφs. Immune cells were cultured with *S. aureus*, *S. pneumoniae*, and *P. aeruginosa* (A, C, and D, MOI = 10; B, MOI = 1). (**A**) Neutrophils (Neut.) were stimulated with bacteria and the percentage of neutrophils (Ly6G^+^CD11b^+^) undergoing suicidal NETosis (Dead: extracellular dsDNA^+^MPO^+^H3Cit^+^) was quantified by flow cytometry. Each point represents neutrophils isolated from a single mouse (*n* = 3). (**B**) Neutrophil-Mφ coculture (ratio = 1:1) restriction of bacterial growth was quantified by CFU spot plating. Percent growth of bacteria was calculated relative to bacterial growth in the absence of immune cells. Immune cells cultured with bacteria in the presence of DNase (8 U/ml). Each point represents the mean result (biological triplicate) of immune cells isolated from a single mouse (*n* = 3). (**C** and **D**) Cocultures were stimulated with fluorescently labeled bacteria and (C) phagocytosis of bacteria by Mφs, and (D) the level of intracellular lactoferrin within Mφs was quantified by flow cytometry. Immune cells cultured with bacteria in the presence of DNase (8 U/ml). (C) Background MFI was subtracted from each time point, and (D) MFI was normalized by an isotype control. Each point represents immune cells isolated from a single mouse (*n* = 3). Two-way ANOVA with (A) Sidak’s or (B to D) Tukey multiple comparisons test (*P ≤ 0.05, **P ≤ 0.01, ***P ≤ 0.001, and ****P ≤ 0.0001).

Since neutrophils enhance *S. aureus* killing by Mφs through the formation of NETs, we reasoned that this process may occur in response to other NET-inducing bacterial pathogens from different phylogenetic lineages, including *S. pneumoniae* (*66*) and *P. aeruginosa* (*67*). As observed for *S. aureus*, A9^−/−^ neutrophils have accelerated and more robust suicidal NETosis in response to *S. pneumoniae* and *P. aeruginosa* relative to WT (Fig. 9A and fig. S9A). In addition, neutrophil-Mφ cocultures containing A9^−/−^ neutrophils restricted *S. pneumoniae* and *P. aeruginosa* growth better than cocultures containing WT neutrophils (Fig. 9B). DNase treatment prevented the improved restriction of bacterial growth in cocultures containing A9^−/−^ neutrophils, indicating a dependence on NET formation. A similar but smaller restriction in *S. aureus* growth was observed in neutrophil cultures (fig. S9B); however, A9^−/−^ neutrophils did not restrict *P. aeruginosa* growth better than WT despite NETs facilitating killing, and NETs alone were not antibacterial toward *S. pneumoniae* (fig. S9B). DNase treatment did not alter Mφ killing in the absence of neutrophils (fig. S9C). These results suggest that NET formation broadly increases killing of extracellular bacterial pathogens in the presence of Mφs.

NET formation facilitates phagocytosis of *S. aureus* by Mφs (Fig. 7) and transfers neutrophil-specific AMPs to Mφs (Fig. 8). Similarly, phagocytosis of *S. pneumoniae* and *P. aeruginosa* by Mφs was enhanced in cocultures containing A9^−/−^ neutrophils relative to cocultures with WT neutrophils (Fig. 9C and fig. S9D). Further, Mφs in cocultures containing A9^−/−^ neutrophils accumulated higher levels of lactoferrin compared to cocultures with WT neutrophils in response to *S. pneumoniae* and *P. aeruginosa* (Fig. 9D and fig. S9E). DNase treatment reversed both of these phenotypes (Fig. 9, C and D). Thus, NET formation in the presence of Mφs appears to be an immune defense mechanism that broadly enhances antibacterial activity against multiple distinct bacterial pathogens from different phylogenetic lineages.

## DISCUSSION

**Fig. 10.**
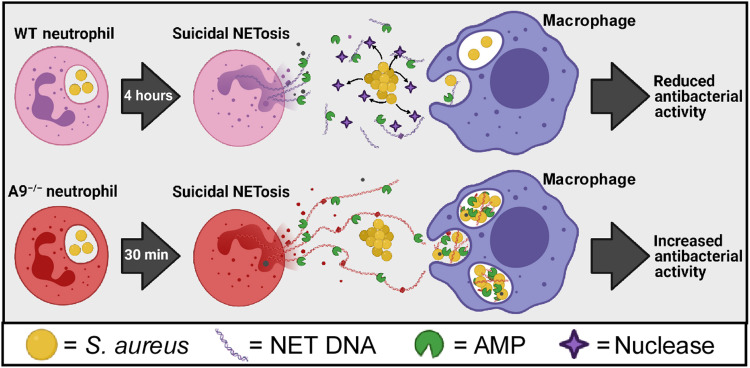
Proposed model for how accelerated NET formation by A9^−/−^ neutrophils enhances Mφ antibacterial activity. Upon engaging *S. aureus*, WT neutrophils undergo suicidal NETosis after 4 hours. However, *S. aureus* secretes a nuclease that degrades NETs and uncouples the cooperation between neutrophils and Mφs during infection. In contrast, A9^−/−^ neutrophils in response to *S. aureus* undergo comparable levels of suicidal NETosis as WT after 30 min. This accelerated and more robust NET formation outpaces/overwhelms *S. aureus*, which allows for increased phagocytosis of *S. aureus* into Mφs, transferring of neutrophil-specific AMPs to Mφs, and increased antibacterial activity. Therefore, NET formation acts as a conduit for neutrophils and Mφs to combat bacterial pathogens cooperatively during infection that is particularly important within the immunological niche of the heart. Created with BioRender.com.

We report that A9^−/−^ neutrophils produce higher levels of mitochondrial O_2_^−^ (Figs. 3 and 4) in response to *S. aureus.* As a result of increased levels of mitochondrial O_2_^−^, A9^−/−^ neutrophils undergo accelerated and robust suicidal NETosis in response to multiple bacterial pathogens (Figs. 2D and 9A), which coincides with increased survival of A9^−/−^ mice during systemic *S. aureus* infection (Fig. 1A). This identifies intracellular S100A9 as a critical molecular rheostat in neutrophil function. Enhanced suicidal NETosis by A9^−/−^ neutrophils does not increase bacterial killing in isolation (Fig. 6A and fig. S9B) but augments killing in the presence of Mφs (Figs. 6B and 9B). NET formation acts as a conduit to increase phagocytosis of multiple phylogenetically distinct bacterial pathogens by Mφs (Figs. 7 and 9C) and transfers neutrophil-specific AMPs to Mφs (Figs. 8 and 9D). By using A9^−/−^ neutrophils as a tool to assess NET-related biology, these results demonstrate that NET formation enables neutrophils to broadly combat extracellular bacterial pathogens cooperatively with Mφs. A graphical model demonstrating how NET formation augments the antibacterial activity of Mφs is provided in Fig. 10.

Our study focused on how a deficiency in S100A9 enhances the antibacterial response, which allowed for the identification of a previously unidentified antibacterial mechanism. While the synergy in bactericidal activity between neutrophils and Mφs was exaggerated when using A9^−/−^ neutrophils, it is important to note that NET formation by WT neutrophils also enhances the antibacterial activity of Mφs (Fig. 6B). However, expression of nuclease by *S. aureus* limits the capacity for WT neutrophils to cooperate with Mφs (Fig. 6D). These findings highlight *S. aureus* as a professional pathogen that is capable of uncoupling cooperation among innate immune cells through NET degradation. In addition, alterations in S100A9 abundance have been associated with many clinically relevant inflammatory states, such as autoimmunity (*68*, *69*), cancer (*70*–*72*), coronavirus disease 2019 (COVID-19) (*73*), and aging (*74*). As a result, changes in the abundance of S100A9 may render patient populations more or less susceptible to bacterial infections.

Calprotectin plays an integral role in nutritional immunity as bacterial burdens were reduced in the kidney and liver of A9^−/−^ mice infected with Newman (*16*, *17*); however, this was not observed in A9^−/−^ mice infected with USA300 (LAC) (Fig. 1B). This difference in infection may be attributed to alterations in virulence (*44*). For example, Sae activation leads to transcription of a wide range of virulence factors, and Newman constitutively expresses the *sae* operon due to an amino acid substitution in the sensor histidine kinase SaeS (*75*). Since Sae activation plays a vital role in responding to phagocyte-related stressors (*76*) upstream of nuclease expression (*52*), this could provide one possible explanation as to why Newman and USA300 show some differences in pathology during infection.

Extracellular functions for calprotectin have been well described (*16*–*21*), but intracellular S100A9 also contributes to disease pathology. Previous studies demonstrate that intracellular S100A9 regulates the respiratory burst (*77*), and we observe that A9^−/−^ neutrophils have a heightened respiratory burst than WT neutrophils in response to *S. aureus* in vitro (fig. S1D). However, A9^−/−^ and WT neutrophils produce comparable levels of ROS in vivo (fig. S1E), suggesting that the alterations to the respiratory burst are likely not accounting for the increased protection of A9^−/−^ mice from systemic *S. aureus* infections. Instead, we provide evidence that intracellular S100A9 regulates mitochondrial homeostasis, whereby A9^−/−^ neutrophils produce higher levels of mitochondrial O_2_^−^ in response to *S. aureus* (Figs. 3 and 4). As a result, A9^−/−^ neutrophils have enhanced suicidal NETosis and decreased primary degranulation (Figs. 2 and 4). Why the heart offers a unique niche for A9^−/−^ neutrophil function is unclear. S100A9 can modulate glycolysis through the nitrosylation of glyceraldehyde phosphate dehydrogenase (*78*); therefore, it is possible that S100A9 directly or indirectly influences mitochondrial homeostasis and the generation of O_2_^−^ from the electron transport chain, thereby linking S100A9 to mitochondrial metabolism. In addition, activation of the inositol-requiring enzyme 1α in response to *S. aureus* promotes heightened mitochondrial ROS production and NETosis (*58*). While inflammatory conditions of heightened endoplasmic reticulum (ER) stress correlate with increased levels of S100A9 (*79*, *80*), whether intracellular S100A9 regulates ER homeostasis is unexplored. While the exact mechanism by which intracellular S100A9 alters mitochondrial homeostasis is unknown, the unique metabolic/immunologic niche of the heart may amplify suicidal NETosis in A9^−/−^ neutrophils during infection. Data presented here demonstrate that A9^−/−^ neutrophils in the heart produce more mitochondrial O_2_^−^ during *S. aureus* infection, thereby enhancing suicidal NETosis, which is beneficial for the host during systemic *S. aureus* infections.

NETs are used to broadly combat bacterial infections. *S. aureus* (*8*, *9*), *Shigella flexneri* (*9*), *S. pneumoniae* (*66*), *Neisseria gonorrhoeae* (*81*), and *P. aeruginosa* (*67*) trigger NETosis; however, the mechanism for the antimicrobial activity of NETs is enigmatic. In Mφs, impaired maturation of the lysosome causes internalized cargo to recycle to and accumulate on the extracellular membrane (*82*). We propose a model where altered phagosomal function in neutrophils results in suicidal NETosis, thereby preventing intracellular bacterial replication and enhancing the antimicrobial activity of Mφs. *S. aureus* can survive the antimicrobial stresses of the phagosome in neutrophils (*83*), which provides *S. aureus* a long-term reservoir to replicate unperturbed (*84*). Suicidal NETosis may offer a mechanism to expel intracellular bacteria from the cell while entrapping the bacteria in AMP-rich NETs. These NETs act as a pseudo-opsonin that heightens phagocytosis of bacteria into Mφs (Figs. 7 and 9) and transfers neutrophil-specific AMPs to Mφs (Fig. 8 and 9). Internalization of AMPs into a phagosome is particularly important because the extracellular release of elastase and many proteases bound to NETs limits their antimicrobial activity (*85*–*89*). Thus, NETs may provide an efficient mechanism to transfer AMPs back to a phagosomal compartment within Mφs and restore the full activity of AMPs, which coincides with NET formation enhancing the killing of bacterial pathogens in the presence of Mφs (Figs. 5 and 9). Similar to nuclease secretion preventing NETs from eliciting direct antibacterial activity on *S. aureus* (*10*), nuclease secretion also can uncouple the NET-mediated cooperation between neutrophils and Mφs (Fig. 6D). We provide evidence that accelerated and more robust NET formation, observed in A9^−/−^ neutrophils, can outpace/overwhelm nuclease activity and increase antibacterial activity in the presence of Mφs.

The transfer of neutrophil-specific AMPs to Mφs is not without precedent. NET formation allows for the transfer of neutrophil elastase to Mφs, which facilitates NET DNA translocation into the cytosol of Mφs and inducing the production of type I interferon by activating cyclic guanosine monophosphate–adenosine monophosphate synthase (cGAS) (*56*). We provide evidence that NETs transfer enzymatically active neutrophil elastase to Mφs in response to *S. aureus* (Fig. 8F). Similarly, in response to intracellular pathogens such as *Legionella pneumophila* (*33*) and *Mycobacterium tuberculosis* (*34*–*36*), phagocytosis of infected apoptotic neutrophils transfers AMPs to Mφs, which enhances Mφ killing. However, apoptotic debris was unable to facilitate the same antibacterial enhancement of Mφs in response to *S. aureus* (Fig. 6E), suggesting that these mechanisms may be pathogen specific. The mechanism by which AMPs are delivered to the Mφs may also be critical to establishing the appropriate immune response. Mφs undergo anti-inflammatory responses following phagocytosis of apoptotic cells (*90*, *91*), whereas necrotic cells (*92*, *93*) and NETs (*94*) induce proinflammatory responses. The molecular events governing whether neutrophils undergo suicidal NETosis or apoptosis may be pathogen specific and play a critical function in regulating inflammation. Our results demonstrate that NET formation acts as a conduit to broadly enhance antimicrobial activity in the presence of Mφs in response to bacterial pathogens.

## MATERIALS AND METHODS

### Mice

C57BL/6J and B6.Cg-*Padi4^tm1.1Kmow^*/J mice were purchased from the Jackson Laboratory (JAX mice stock no. 000664 and no. 030315). B6.*S100a9*^−/−^ mice were maintained in an accredited animal facility. Mice were housed two to five to a cage in specific pathogen–free conditions and randomly assigned to experimental groups. Food and water were provided ad libitum. All animal experiments were approved and performed in compliance with the Vanderbilt Institutional Animal Care and Use Committee.

### Reagents

Antibodies specific for Ly6G, F4/80, CD35, and rabbit immunoglobulin G (IgG) were from BioLegend; CD11b was from Tonbo; CD63 was from BD Biosciences; and MPO, H3Cit, lactoferrin, PR3, neutrophil elastase, Fas, dsDNA, and mouse IgG alkaline phosphatase were from Abcam. Streptavidin Alexa Fluor 488 was from BioLegend. Live/dead stain and DHR123, CellTracker Orange, CellTrace Violet, CellMask Deep Red, and BacLight Green Bacterial Stain were from Invitrogen; Helix NP Blue was from BioLegend. Murine Fc-blocking antibody (2.4G2) was from Tonbo. PMA and DNase I were ordered from Sigma-Aldrich. Cytochalasin D was purchased from Cayman Chemical. Paraformaldehyde was from Electron Microscopy Sciences. The neutrophil elastase activity kit was purchased from Abcam. Histopaque 1119 and Histopaque 1077 were from Sigma-Aldrich; Lympholyte was from Cedarlane Laboratories. Dulbecco’s modified Eagle’s medium (DMEM) and phosphate-buffered saline (PBS) that were from Gibco and fetal bovine serum (FBS) from Atlanta Biologicals were used for all tissue culture.

### Bacterial strains

All *S. aureus* experiments used the bacterial strain USA300 (LAC) (*95*). The *nuc-*deficient mutant and control strains were previously described (*96*). The *agr::tet* and control strain was previously described (*55*). The *S. pneumonia* bacterial strain was TIGR4 (*97*) and the *P. aeruginosa* strain was PAO1 (*98*). Bacteria were maintained as a −80°C stock, and *S. aureus* streaked onto tryptic soy agar (TSA; 2% agar), *S. pneumoniae* streaked onto TSA with 5% sheep blood, and *P. aeruginosa* streaked onto Luria-Bertani agar (LB; 2% agar) plates 2 days before each experiment. *S. aureus* and *P. aeruginosa* plates were grown overnight at 37°C, and the *S. pneumoniae* plate was grown overnight (37°C, 5% CO_2_). Single colonies of *S. aureus* and *P. aeruginosa* were transferred to tryptic soy broth (TSB) or LB broth, and liquid cultures were grown overnight at 37°C with 180 rpm of shaking. The entire *S. pneumoniae* plate was transferred to TSB in a sealed 15-ml conical, and liquid cultures were grown overnight at 37°C. For all in vitro experiments, overnight cultures were diluted 1:10 into non–heat-inactivated FBS for 2 hours on ice before coculture with neutrophil unless otherwise stated.

### Infections

Retro-orbital infections were performed as described (*99*). *S. aureus* USA300 was streaked from frozen stocks onto TSA 2 days before infection. Overnight cultures were grown in TSB and then subcultured 1:100 into 5 ml of TSB. *S. aureus* was grown to the mid-exponential phase, washed, and resuspended in 100 μl of ice-cold PBS. All infections used 10- to 13-week-old female mice, which were anesthetized with intraperitoneal injection of 2,2,2-tribromoethanol diluted in PBS. Systemic infections were induced by intravenous injection of the retro-orbital sinus with 2 × 10^7^ or 5 × 10^7^ colony-forming units (CFU) of USA300. Mice were monitored before humane euthanasia by inhalation of CO_2_ at 4 dpi. For MitoTEMPO treatments, the drugs were diluted in sterile PBS and administered by intraperitoneal injection (0.7 mg/kg) 24 hours before infection and every 24 hours thereafter.

### Organ harvest for CFU and neutrophil characterization

Single-cell suspensions were created from the tissue of hearts, livers, and kidneys by processing the organs through a 35-μm filter into fluorescence-activated cell sorter (FACS) media [PBS, 2% (v/v) FBS, and 0.02% NaAz]. Heart tissue was cut into small pieces using scalpels to facilitate processing of the tissue through the filter, and special care was taken to flush out the blood from the heart chambers to ensure that isolated immune cells are from the tissue. Aliquots from each organ homogenate were removed for CFU spot plating. Organ homogenates were pelleted, and the supernatant was removed to quantify NET abundance for ELISA. The remaining organ homogenate was red blood cell–lysed per the manufacturer’s instructions (BioLegend) and divided into separate aliquots in FACS media to be stained ex vivo for flow cytometry.

### Neutrophil isolation

Single-cell suspensions of bone marrow were prepared from the tibias and femurs of mice. Polymorphonuclear granulocytes were isolated using density centrifugation where the neutrophils were isolated at the interface between the layers of Histopaque 1119 and Histopaque 1077. Neutrophils were rested on ice for at least 1 hour in D10 media [DMEM + 10% (v/v) FBS] and then transferred to ultralow-cluster round-bottom 96-well plates (Costar) in D10 media and incubated (37°C, 5% CO_2_) for 1 hour before experiments. The isolated cells were 85 to 95% neutrophils (CD11b^+^Ly6G^+^).

### Bone marrow Mφ cultures

To differentiate bone marrow Mφs (BMMφs), we generated the L929 cell (European Collection of Cell Cultures) supernatant by plating 2.5 × 10^5^ cells into a T150 flask with 50 ml of D10 media and by incubating for 12 days (37°C, 5% CO_2_). After 12 days, the supernatant was collected and sterile filtered.

BMMφs were derived following procedures that have been previously described (*82*). Bone marrow was flushed from the tibias and femurus of mice, and single-cell suspensions were created. Lympholyte separation medium (Cedarlane Laboratories) was used to isolate mononuclear cells, which were plated in a 60-mm petri dish with 6 ml of BMMφ differentiation media [D10 media with 10% (v/v) L-cell supernatant]. Cells were cultured overnight (37°C, 5% CO_2_), and nonadherent cells were transferred onto non–tissue culture–treated 100-mm petri dishes (1-ml cells per petri dish) with 7 ml of BMMφ differentiation media. Cells were incubated for 6 days (37°C, 5% CO_2_) with an additional supplementation of 5 ml of BMMφ differentiation media on day 4 to promote BMMφ differentiation. On day 7, ice-cold PBS was used to remove BMMφs from the dish. BMMφ cultures were 98% CD11b^+^, I-A^lo^, and B7.2^lo^.

### Recombinant calprotectin

Recombinant S100A8 and S100A9 were isolated as described (*100*). Murine S100A8 and S100A9 in pQE32 expression vectors (a gift of C. Kerkhoff) were transformed separately into C41 (DE3) *Escherichia coli* cells. Cells were induced at 37°C, OD_600_ (optical density at 600 nm) with the addition of 1 mM isopropyl thio-β-d-galactoside and allowed to grow 4 hours after induction and then harvested via centrifugation (6.5 krpm, 20 min, 4°C). Cell pellets were combined and resuspended in lysis buffer A [50 mM tris (pH 8.0), 100 mM NaCl, 1 mM EDTA, 1 mM phenylmethylsulfonyl fluoride, 0.5% Triton X-100, and 10 mM β-mercaptoethanol (BME)]. Once homogenized, the sample was sonicated (10 min, 50 W, 5-s on/10-s off) and centrifuged (20,000 rpm, 20 min). The supernatant was discarded, and the pellet was resuspended in lysis buffer A, sonicated, and spun down. The supernatant was discarded, and the pellet was resuspended in lysis buffer B [50 mM tris (pH 8.0), 100 mM NaCl, and 4 M guanidine hydrochloride], sonicated, and spun down. The supernatant was dialyzed into 4 liters of dialysis buffer [20 mM tris (pH 8.0) and 10 mM BME]. Then, the sample was filtered and loaded onto a SourceQ column (flow rate = 1 ml/min). After loading, the column was washed 3 times with buffer A [20 mM tris (pH 8.0) and 10 mM BME] and eluted with a gradient (10 CV, 0 → 100%) to buffer B [20 mM tris (pH 8.0), 1 M NaCl, and 10 mM BME]. Relevant fractions according to SDS–polyacrylamide gel electrophoresis (PAGE) were pooled, concentrated, and loaded onto a S75 gel filtration column. The column was eluted with 1 CV of S75 buffer [20 mM tris (pH 8.0), 100 mM NaCl, and 10 mM BME]. Relevant fractions were pooled according to SDS-PAGE, flash-frozen, and stored at −80°C. The ToxinEraser Endotoxin Removal Kit (GenScript) was used to remove lipopolysaccharide following the manufacturer’s protocol.

### Flow cytometry

All data were collected using a BD LSRII flow cytometer with FACSDiva software and analyzed using FlowJo (FlowJo LLC, Ashland, OR). For all flow cytometry, samples were gated side scatter height (SSC-H) by side scatter area (SSC-A) followed by forward scatter height (FSC-H) by forward scatter area (FSC-A) to remove doublet populations. The singlet population was gated SSC-A by FSC-A to isolate the granulocyte and/or Mφ populations (representative gating in fig. S1B). The resulting cell population was then assessed for assay-specific fluorescent markers as described below.

#### Coculture staining

For the B6-PAD4^−/−^ neutrophil culture, PAD4^−/−^ neutrophils were stained in 1 μM CellTracker Orange in PBS for 20 min at room temperature and washed in D10 media before adding to unstained B6 neutrophils (representative gating to distinguish cell populations in fig. S2G). For the neutrophil-Mφ cocultures, neutrophils were stained in 5 μM CellTrace Violet, and Mφs were stained in 1 μM CellTracker Orange in serum-free DMEM for 20 min at room temperature and washed in D10 media before combining the cells into a single well at a 1:1 ratio (representative gating to distinguish cell populations in Fig. 8F).

#### Exogenous treatments

Cytochalasin D (10 μg/ml) was added 1 hour, rotenone (0.5 μM) 15 min, and MitoTEMPO (0.5 μM) 2 hours before stimulation with *S. aureus*. When treating Mφs with cytochalasin D in coculture, Mφs were pretreated and thoroughly washed in PBS before the addition of neutrophils. DNase (8 U/ml) or recombinant calprotectin trackers (25 μg/ml) were added concurrently with *S. aureus*.

#### Degranulation flow

Single-cell suspensions isolated from the tissue or isolated neutrophils and/or derived Mφs (referred to as cells for the remainder of the flow cytometry section) were stained with a live/dead dye to monitor membrane integrity 20 min before fixation. Cells were fixed in 4% paraformaldehyde at room temperature and incubated for 15 min at 4°C. Cells were pelleted, aspirated, and resuspended in Fc block for 30 min at 4°C in FACS media. Cells washed and stained with anti-CD11b, anti-Ly6G, anti-CD63 (primary), and anti-CD35 (secretory) or appropriate isotype controls in FACS media for 30 min at 4°C. Following staining, cells were washed and resuspended in FACS media for analysis. Cells were gated for live cells (Live/Dead-negative) and then neutrophils (CD11b- and Ly6G-positive) (representative gating in fig. S1B). The median fluorescence intensity (MFI) was quantified for surface CD63 and CD35 relative to staining with an isotype control antibody.

#### NETosis flow

Cells were live/dead-stained as described above except Helix NP Blue (2.5 nM) was also introduced to the cultures. Cells were fixed, blocked, and stained for neutrophils (anti-CD11b and anti-Ly6G) as described above. While being stained for neutrophils, cells were also stained with anti-MPO and anti-H3Cit antibodies. Cells were washed and stained with fluorescent anti-rabbit IgG and streptavidin in FACS media for 30 min at 4°C. Following staining, cells were washed and resuspended in FACS media for analysis. Cells were gated for neutrophils and then live or dead cells. Neutrophils with permeabilized cell membranes that are positive for extracellular dsDNA, MPO, and H3Cit were defined as having undergone suicidal NETosis, and neutrophils positive for extracellular dsDNA, MPO, and H3Cit and with intact cell membranes were defined as having undergone vital NETosis (representative gating in fig. S2A). The percentage of neutrophils undergoing vital or suicidal NETosis was quantified relative to the total number of neutrophils.

#### Total ROS or mitochondrial O_2_^−^ flow

Cells were live/dead-stained as described in the “Degranulation flow” section except DHR123 (3 μM) was also introduced to the cultures to quantify total ROS production, or MitoSOX red (5 μM) and MitoTracker deep red (500 nM) were introduced to quantify mitochondrial O_2_^−^ production. Cells were fixed, blocked, and stained for neutrophils (anti-CD11b and anti-Ly6G) as described in the “Degranulation flow” section. Following staining, cells were washed and resuspended in FACS media for analysis. Cells were gated for live cells and then neutrophils (representative gating in fig. S1B). For total ROS production, the DHR123 MFI was quantified relative to unstimulated neutrophils (in vitro assays) or mock-treated mice (in vivo infections). For mitochondrial O_2_^−^, MitoSOX MFI was normalized to MitoTracker MFI.

#### Phagocytosis

For in vitro assays, BacLight-labeled bacteria were used, and, for in vivo infections, a fluorescent strain of *S. aureus* (p*SarA_sfGFP*) (*65*) was used to quantify internalized bacteria in vivo. Cells were live/dead-stained, fixed, blocked, and stained for neutrophils (anti-CD11b and anti-Ly6G) as described in the “Degranulation flow” section. If phagocytosis is being quantified in cocultures, then neutrophils and Mφs were distinguished by the staining described in the “Coculture staining” section. If phagocytosis is being quantified in Mφs in vivo, then Mφs were identified as CD11b^+^F4/80^+^. Following staining, cells were washed and resuspended in FACS media for analysis. Cells were gated for live cells and then neutrophils (representative gating in fig. S1B) or Mφs. The MFI was quantified for bacteria per cell, and background fluorescence was subtracted using unstimulated neutrophils or Mφs (in vitro assays) or mock-treated mice (in vivo infections).

#### AMP transfer

Cells were live/dead-stained, fixed, and blocked as described in the “Degranulation flow” section. Neutrophils and Mφs were distinguished by the staining described in the “Coculture staining” section. Following block, cells were washed and stained with anti-lactoferrin, anti-PR3, anti-elastase, or isotype control antibodies in permeabilization buffer (PBS + 0.05% saponin + 0.5% bovine serum albumin) for 30 min at 4°C, washed, and stained with anti-rabbit IgG in permeabilization buffer for 30 min at 4°C. Following all staining, cells were washed and resuspended in FACS media for analysis. Cells were gated for live cells and then neutrophils (representative gating in fig. S1B) or Mφs. The MFI was quantified for intracellular lactoferrin, PR3, and elastase relative to staining with an isotype control antibody.

### Confocal imaging

For NETosis imaging, neutrophils were stained in with CellMask Deep Red in PBS for 20 min at room temperature and washed in D10 media before plating. Neutrophils were incubated for 2 hours (37°C, 5% CO_2_) on glass-bottom petri dishes (MatTek Corporation, Ashland, MA) in phenol red–free D10 to allow cells to properly attach to the dish. For NETosis imaging, *S. aureus* was stained with BacLight Green for 30 min on ice and added to the dish at a multiplicity of infection (MOI) of 1. Helix NP Blue was added 20 min before fixation to stain for extracellular DNA (NET structures). For superoxide imaging, unstained *S. aureus* was added to the dish at an MOI of 1. MitoTracker Deep Red and MitoSOX Red were added 20 min before fixation to stain for mitochondrial structures and superoxide. Cells were fixed in 4% paraformaldehyde at room temperature and incubated for 15 min at 4°C. Dishes were aspirated, and samples were preserved using the ProLong Gold Antifade Mountant. All microscopy was done using a Zeiss 880 microscope with AiryScan and Zeiss Zen Software. Images were analyzed using ImageJ.

### NET DNA ELISA

Vinyl 96-well plates were charged using 0.01% poly-l-lysine solution (Sigma-Aldrich). For samples, wells were coated with anti-MPO or anti-neutrophil elastase antibody, and for the DNA standard, dsDNA (Sigma-Aldrich) was titrated using fourfold dilutions starting at 10 mg/ml. DNA in the wells was detected using an anti-dsDNA antibody (mouse IgG) followed by a secondary anti-mouse antibody conjugated with alkaline phosphatase and *p*-nitrophenyl phosphate (1 mg/ml) (Sigma-Aldrich). Abundance of NET DNA was quantified using a BioTek plater reader (OD = 405λ). Samples and standards were read in duplicate, and DNA abundance was quantified relative to the standard.

### Bacterial killing assay

Neutrophils, Mφs, or neutrophil-Mφ (1:1) cocultures were cultured with bacteria at an MOI of 1 (20,000 CFU per 20,000 immune cells) in D10 (37°C, 5% CO_2_). After 30 min, 2, 6, 12, and 24 hours, samples were serially diluted and spot-plated onto TSA. Percent growth was quantified by dividing the CFU of the bacteria–immune cell cultures by the bacteria alone cultures.

### NET isolation

NETs were isolated as previously described (*61*). Briefly, neutrophils were transferred to ultralow-cluster flat round-bottom six-well plates (Costar) in 0.75 ml of D10 media (1 × 10^6^ cells per well) and incubated (37°C, 5% CO_2_) for 1 hour. To induce NETosis, neutrophils were stimulated with PMA (100 nM) and incubated (37°C, 5% CO_2_) for 4 hours. To induce apoptosis, neutrophils were stimulated with an agonistic anti-Fas antibody (100 ng/ml) and incubated (37°C, 5% CO_2_) for 16 hours. In the final 15 min, the restriction enzyme Alu I (New England Biolabs) was added to the neutrophil cultures (4 U/ml). The supernatant was collected and centrifuged (300*g*) for 5 min at 4°C to remove whole cells and debris. A fraction of the supernatant was stained with Helix NP, and abundance of fluorescent NET structures was quantified in a plate reader to assure equal concentrations of NETs. Overnight *S. aureus* cultures were diluted 1:10 into 500 μl of the NET-rich supernatants and placed on ice for 15 min.

### Neutrophil elastase activity assay

Fluorescently labeled neutrophils and Mφs (as described in the “Coculture staining” section) in coculture were stimulated with *S. aureus* (MOI = 10). After 2 hours, immune cells were pelleted and resuspended in ice-cold FACS media for fluorescence-activated cell sorting. Isolated immune cells were pelleted and resuspended in lysis buffer (1% IGEPAL + protease inhibitors). Lysates were held on ice for 5 min followed by the removal of particulate material by centrifugation at 12,000*g* for 10 min at 4°C. Neutrophil elastase activity was detected per the manufacturer’s instructions (Abcam) using a BioTek Cytation 5 fluorescence plate reader.

### Nuclease activity assay

In rhodamine-free D10 media, 5 × 10^6^ CFU of bacteria were stimulated with 30 μM calprotectin in a round-bottom 96-well plate (37°C, 5% CO_2_) to stimulate the Sae sensory system. At appropriate time points, half of the supernatant from the bacteria was transferred to a flat-bottom, black-wall, 96-well plate containing calf thymus DNA stained with Helix NP in PBS (500 ng/ml). A titration of DNase I was used a standard to quantify relative nuclease activity. Fluorescence intensity (excitation: 440 ± 10, emission: 480 ± 10) was read every 15 min in a plate reader at 37°C with shaking. The decay rate of fluorescence was normalized to account for photobleaching (labeled DNA + supernatant from wells containing no bacteria but stimulated with calprotectin), and relative activity was quantified by fitting the kinetic curves to the DNase I standard. The presence of calprotectin in supernatants from wells lacking bacteria had no effect on DNA degradation. The remaining bacteria in the first plate were resuspended, and OD_600_ was quantified in a plate reader to normalize nuclease activity to bacterial concentrations.

### Statistics

Specific statistical details for each experiment can be found in the corresponding figure legends. Error bars for all experiments represent SEM across mice. A minimum of three experimental replicates were performed for each assay, and the specific number of replicates is noted in the corresponding figure legend. All *P* values were calculated using a two-way analysis of variance (ANOVA) (with Sidak’s multiple comparisons test or Tukey multiple comparisons test), unpaired *t* test, or log-rank (Mantel-Cox) test when applicable. Statistical work was performed using Prism 6 software (GraphPad), and significance is indicated on the graphs as follows: *P ≤ 0.05, **P ≤ 0.01, ***P ≤ 0.001, and ****P ≤ 0.0001; ns, not significant.

## Notes

View/request a protocol for this paper from *Bio-protocol*.
