# Supplementary material for: Neutrophil extracellular traps enhance macrophage killing of bacterial pathogens
Source: Sci Adv. 2021 Sep 10;7(37):eabj2101. doi: 10.1126/sciadv.abj2101 (PMC8442908; doi:10.1126/sciadv.abj2101)
Supplement: Supplementary file 1 — Figs. S1 to S9 [file sciadv.abj2101_sm.pdf]

Science Advances
AAAS

Supplementary Materials for

Neutrophil extracellular traps enhance macrophage killing of bacterial pathogens

Andrew J. Monteith, Jeanette M. Miller, C. Noel Maxwell, Walter J. Chazin, Eric P. Skaar*

*Corresponding author. Email: eric.skaar@vumc.org

Published 10 September 2021, *Sci. Adv.* **7**, eabj2101 (2021)
DOI: 10.1126/sciadv.abj2101

This PDF file includes:

Figs. S1 to S9

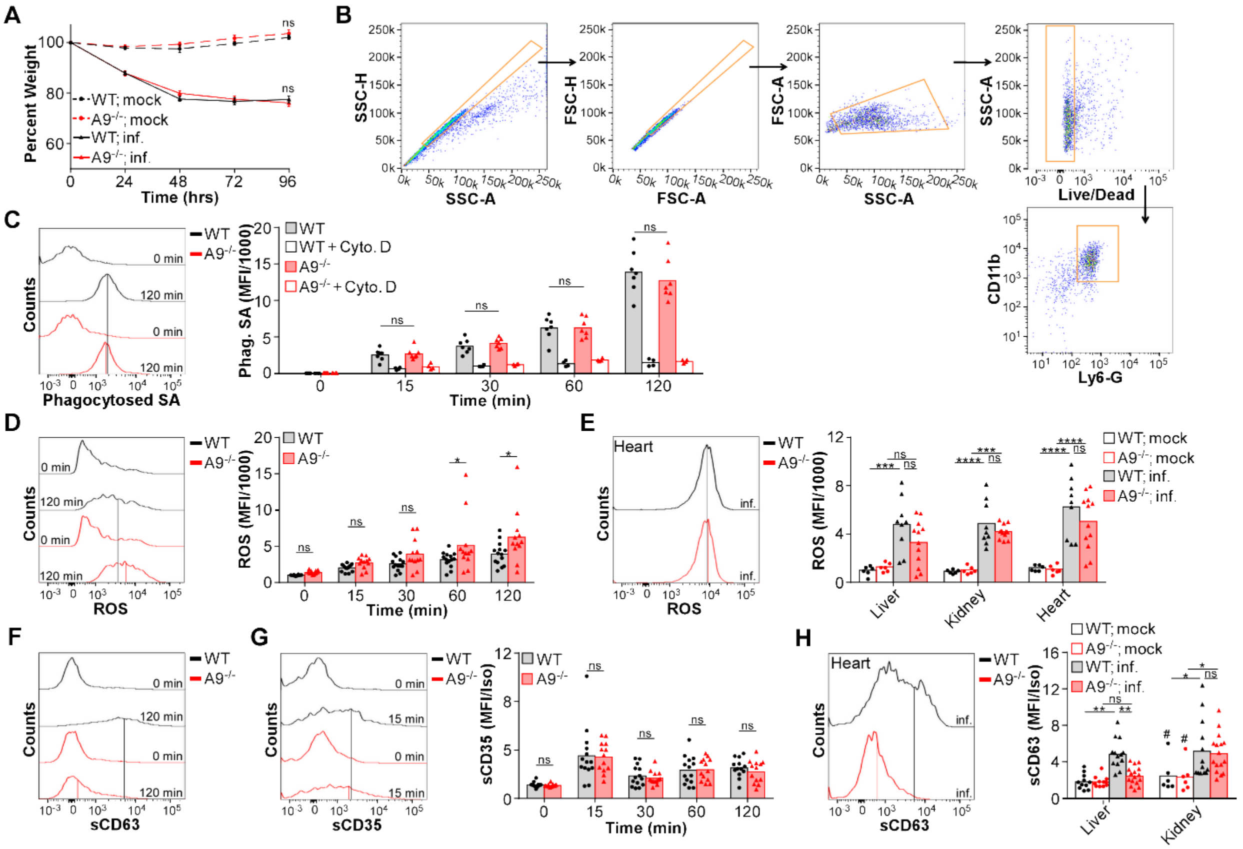

Fig. S1. A9$^{-/-}$ neutrophils have comparable phagocytosis and ROS production. (**A**) Mice were systemically infected (inf.) with USA300 (CFU=2x10^{7}) and weight loss was monitored during infection. Percent weigh loss is normalized to starting weight. Each point represents the mean of all mice ±standard error (mock n=14, WT; inf. n=31, A9$^{-/-}$; inf. n=29). (**B**) The representative gating scheme identifying neutrophils (Ly6G^{+}CD11b^{+}) by flow cytometry is provided. (**C-D**) Neutrophils were cultured with *S. aureus* (MOI=10) and (*C*) phagocytosis of fluorescent *S. aureus* by neutrophils and (*D*) total ROS production by neutrophils was quantified by flow cytometry. (*C*) Neutrophils were pretreated with cytochalasin D (Cyto. D; 10 µg/mL) for 1 hr prior to adding *S. aureus* to prevent internalization. (*C*) Background median fluorescent intensity (MFI) subtracted from each time point or (*D*) MFI normalized to unstimulated WT. Each point represents neutrophils isolated from a single mouse (*C*, untreated n=7, Cyto. D treated n=4; *D*, WT n=14, A9$^{-/-}$ n=13). (**E**) Mice were systemically infected with *S. aureus* (CFU=2x10^{7}). 4 dpi, organs were homogenized

and total ROS production by neutrophils was quantified by flow cytometry. MFI normalized to uninfected WT. Each point represents a single mouse (mock n=11, WT; inf n=13, A9$^{-/-}$; inf n=16). (**F-G**) Neutrophils were cultured with *S. aureus* (MOI=10) and (*F*) primary (surface CD63; sCD63) and (*G*) secretory (surface CD35; sCD35) degranulation by neutrophils were quantified by flow cytometry. (*G*) MFI normalized by an isotype control. Each point represents neutrophils isolated from a single mouse (WT n=14, A9$^{-/-}$ n=13). (**H**) Mice were systemically infected with *S. aureus* (CFU=2x10^{7}). 4 dpi, organs were homogenized and primary degranulation by neutrophils was quantified by flow cytometry (#=not enough events in uninfected organs to accurately quantify MFI for some of the mice). MFI normalized by an isotype control. Each point represents a single mouse (mock n=11, WT; inf. n=13, A9$^{-/-}$; inf. n=16). (*C-H*) Vertical line on histogram is MFI. Two-way ANOVA with (*C, E, H*) Tukey or (*D, G*) Sidak's multiple comparisons test (**P*≤0.05, ***P*≤0.01, ****P*≤0.001, *****P*≤0.0001, ns=not significant).

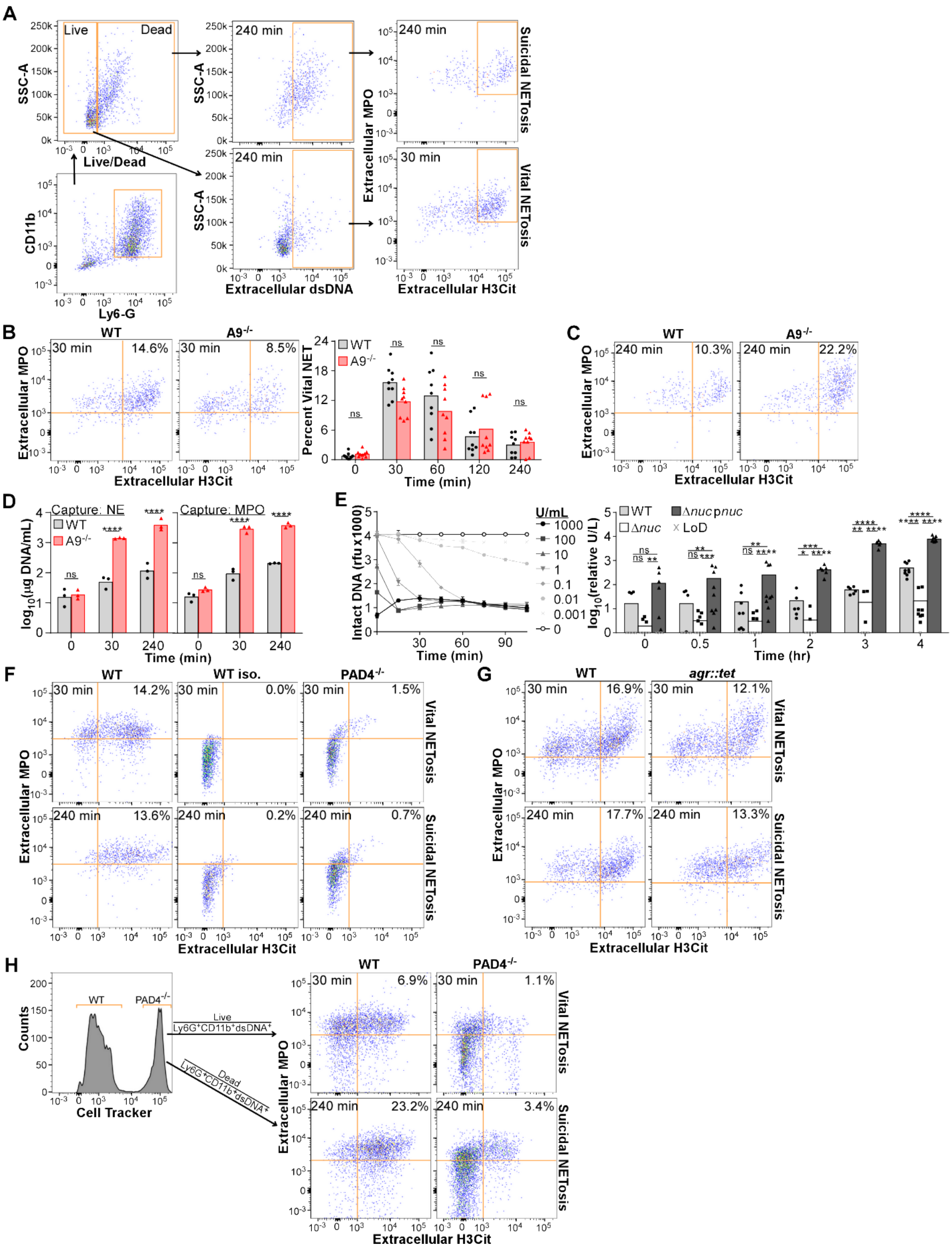

Fig. S2. Vital NETosis is comparable in WT and A9$^{-/-}$ neutrophils in response to *S. aureus*.

(**A-D**) Neutrophils were cultured with *S. aureus* (MOI=10). (*A*) The representative flow gating scheme identifying vital and suicidal NETosis by flow cytometry is provided. Data is from WT neutrophils and quantified in panels *B-C*. (*B-C*) The percentage of neutrophils ($Ly6G^+CD11b^+$) undergoing (*B*) vital (Live: extracellular $dsDNA^+MPO^+H3Cit^+$) or (*C*) suicidal (Dead: extracellular $dsDNA^+MPO^+H3Cit^+$) NETosis in response to *S. aureus* were quantified by flow cytometry. (*B*) Each point represents neutrophils isolated from a single mouse (n=9). (*D*) Supernatant from neutrophils stimulated with *S. aureus* were used to quantify NET abundance in the culture by ELISA. Capture antibodies directed towards MPO or neutrophil elastase (NE). NET DNA abundance was quantified using a DNA standard. Each point represents the mean result (biological duplicate) from supernatants isolated from neutrophils from a single mouse (n=3). (**E**) The Sae sensory system in *S. aureus* was stimulated with recombinant calprotectin (30 μM) for the indicated amount of time to induce nuclease secretion and isolated supernatants were transferred to wells containing fluorescently labeled DNA. Degradation of fluorescently labeled DNA was quantified kinetically in a plate reader. Units (U) of activity is relative to a standard curve using DNase I (Limit of detection=LoD). Each point represents the mean results (biological duplicate) of 6-9 overnight cultures (n=6-9). (**F-H**) Neutrophils were cultured with *S. aureus* (MOI=10) and the percentage of neutrophils undergoing vital (Live: extracellular $dsDNA^+MPO^+H3Cit^+$) or suicidal (Dead: extracellular $dsDNA^+MPO^+H3Cit^+$) NETosis in response to *S. aureus* were quantified by flow cytometry. (*F*) Representative NETosis flow plots contrasting WT and $PAD4^{-/-}$ neutrophils stained with anti-MPO and -H3Cit antibodies and WT neutrophils stained with isotype (iso.) control antibodies. (*G*) Representative NETosis flow plots of WT neutrophils responding to WT and *agr*-deficient (*agr::tet*) strains of *S. aureus.* (*H*) Representative NETosis flow plots of co-cultures containing WT (Cell Tracker negative) and $PAD4^{-/-}$ (Cell Tracker positive) neutrophils. Two-way ANOVA with (*B, D*) Sidak's or (*E*) Tukey multiple comparisons test (**P*≤0.05, ***P*≤0.01, ****P*≤0.001, *****P*≤0.0001, ns=not significant).

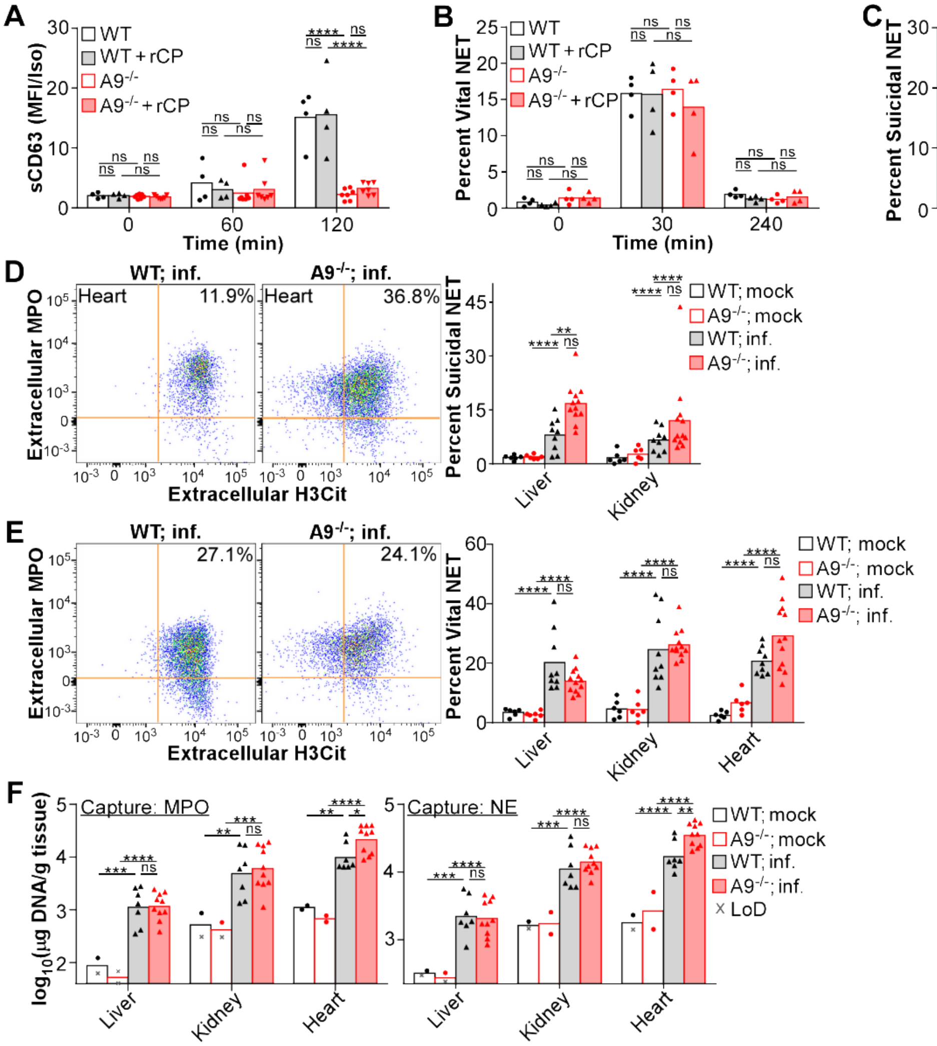

Fig. S3. Addition of recombinant calprotectin does not alter the response of A9$^{-/-}$ neutrophils to *S. aureus*. (**A-C**) Neutrophils were cultured with *S. aureus* (MOI=10) and recombinant calprotectin (rCP; 25 µg/mL). (*A*) Primary degranulation (sCD63) by neutrophils (Ly6G^{+}CD11b^{+}) and percentage of neutrophils undergoing (*B*) vital (Live: extracellular dsDNA^{+}MPO^{+}H3Cit^{+}) and (*C*) suicidal (Dead: extracellular dsDNA^{+}MPO^{+}H3Cit^{+}) NETosis was quantified by flow cytometry. (*A*) MFI was normalized by an isotype control. Each point represents immune cells isolated from a single mouse (n=4). (**D-F**) Mice were systemically infected (inf.) with *S. aureus* (CFU=2x10^{7}). 4 dpi, organs were homogenized and the percentage of neutrophils undergoing (*D*) vital and (*E*) suicidal NETosis were quantified by flow cytometry. Each point represents a single mouse (mock n=6, WT; inf. n=9, A9$^{-/-}$; inf. n=12). (*F*) Supernatant from

homogenized organs were used to quantify NET abundance in the tissues by ELISA. Capture antibodies directed towards MPO or neutrophil elastase (NE). NET DNA abundance was quantified using a DNA standard and normalized to the mass of the tissue (Limit of detection=LoD). Each point represents the mean result (biological duplicate) from supernatants isolated from a single mouse (mock n=2, WT; inf. n=7, A9$^{-/-}$; inf. n=10). (*A*-*F*) Two-way ANOVA with Tukey multiple comparisons test (**P*≤0.05, ***P*≤0.01, ****P*≤0.001, *****P*≤0.0001, ns=not significant).

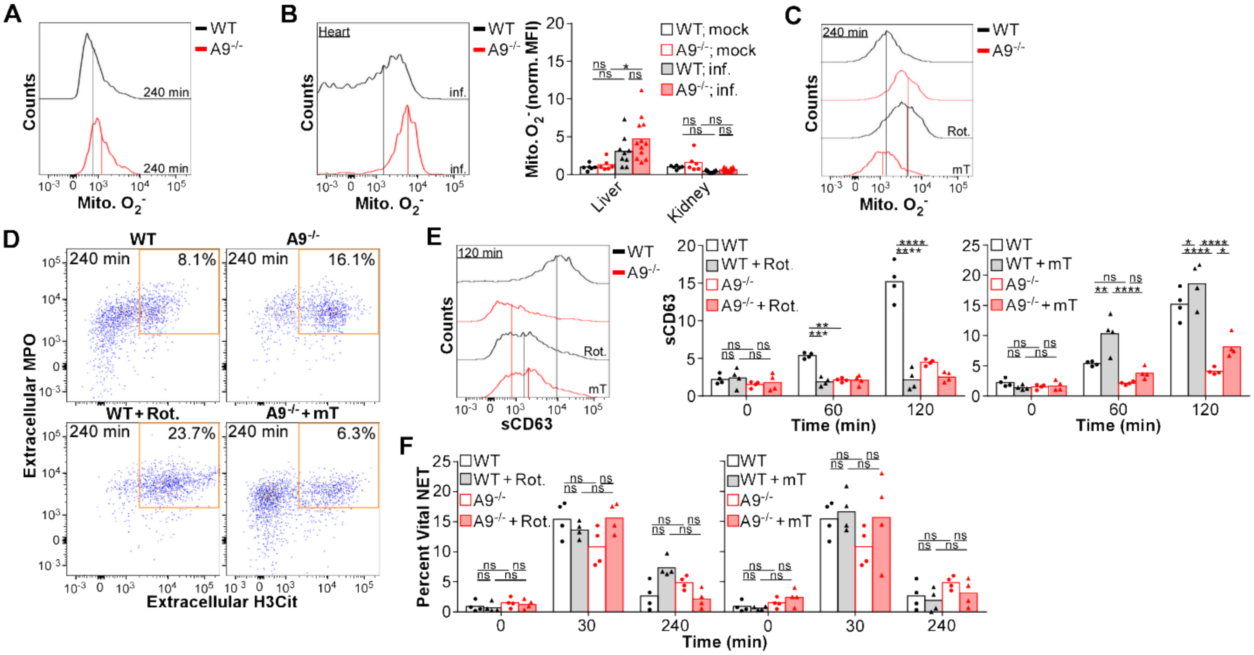

Fig. S4. Mitochondrial superoxide production suppresses primary degranulation in response to *S. aureus*. (**A**) Neutrophils were cultured with *S. aureus* (MOI=10). Mitochondrial O_2^- by neutrophils ($Ly6G^+CD11b^+$) was quantified by flow cytometry. (**B**) Mice were systemically infected (inf.) with USA300 (CFU=$2x10^7$). 4 dpi, organs were homogenized and mitochondrial O_2^- production by neutrophils in the tissues were quantified by flow cytometry. mitoSOX MFI was normalized by mitoTracker MFI. Each point represents a single mouse (mock n=6, WT; inf n=9, $A9^{-/-}$; inf n=12). (**C-F**) Neutrophils were pretreated with rotenone (Rot.; 0.5 μM) for 15 min or mitoTEMPO (mT; 0.5 μM) for 2 hr were cultured with *S. aureus* (MOI=10). (*C*) Mitochondrial O_2^- production by neutrophils, (*D, F*) the percentage of neutrophils undergoing (*D*) suicidal (Dead: extracellular $dsDNA^+MPO^+H3Cit^+$) or (*F*) vital (Live: extracellular $dsDNA^+MPO^+H3Cit^+$) NETosis, and (*E*) the level of primary degranulation (sCD63) was quantified by flow cytometry. (*E*) MFI normalized by an isotype control. (*E-F*) Each point represents immune cells isolated from a single mouse (n=4). (*A-C, E*) Vertical line on histogram is MFI. (*B, E-F*) Two-way ANOVA with Tukey multiple comparisons test (**P*≤0.05, ***P*≤0.01, *****P*≤0.0001, ns=not significant).

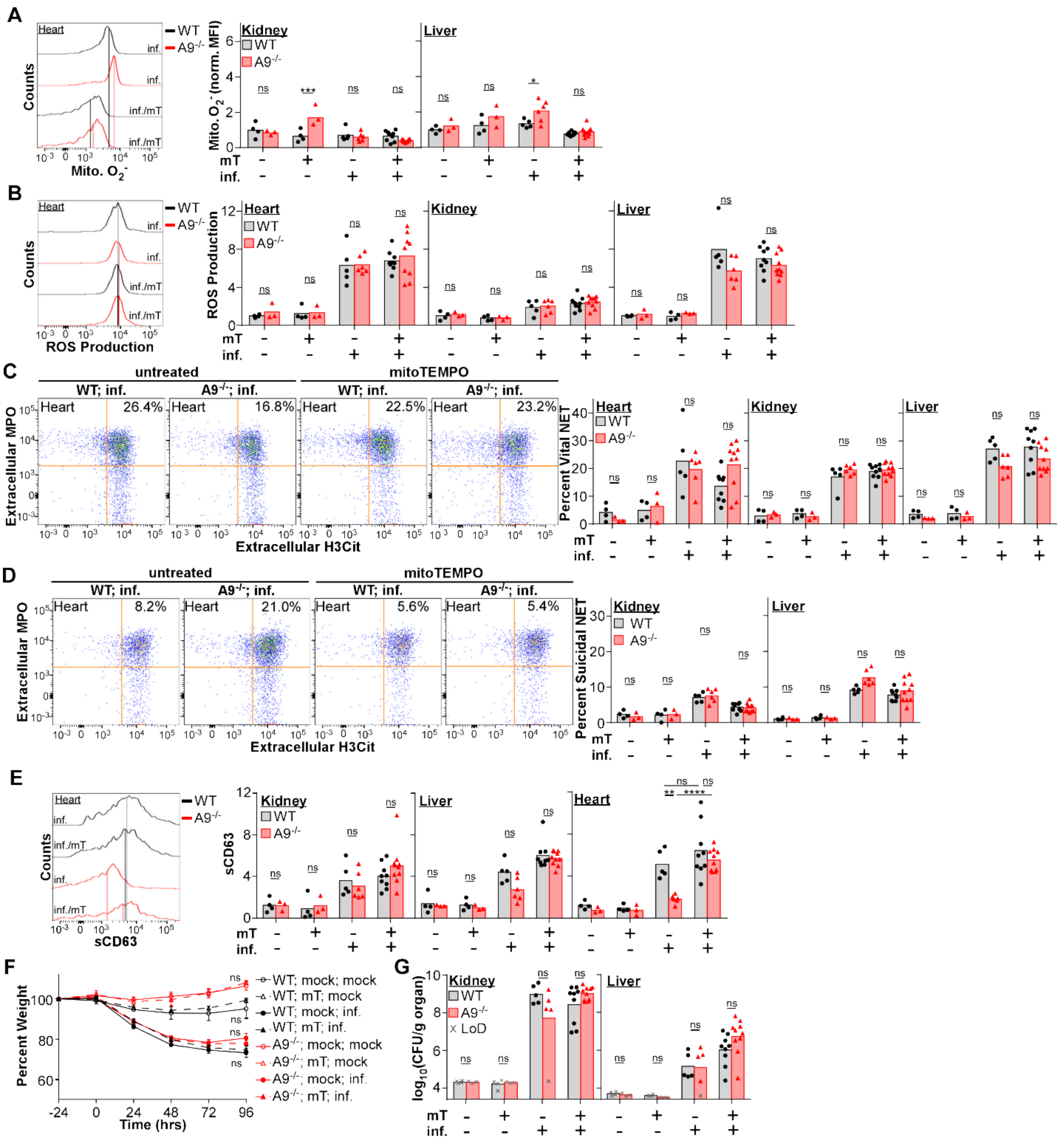

Fig. S5. mitoTEMPO treatment reduces mitochondrial O_2^- in neutrophils during infection. (A-G) Mice were treated with mitoTEMPO (mT; 0.7 mg/kg) by intraperitoneal injection 24 hr prior to infection and every 24 hr during infection. Mice were systemically infected (inf.) with *S. aureus* (CFU=2x10^7). (*A-E*) 4 dpi, organs were homogenized and (*A*) mitochondrial O_2^- and (*B*) total ROS production by neutrophils (Ly6G$^+$CD11b$^+$), (*C-D*) percentage of neutrophils undergoing (*C*) vital

(Live: extracellular dsDNA$^+$MPO$^+$H3Cit$^+$) and (*D*) suicidal (Dead: extracellular dsDNA$^+$MPO$^+$H3Cit$^+$) NETosis, and (*E*) primary degranulation (sCD63) by neutrophils were quantified by flow cytometry. (*A*) mitoSOX MFI normalized by mitoTracker MFI, (*B*) MFI normalized to uninfected WT, and (*E*) MFI normalized by an isotype control. Each point represents a single mouse (mock; mock/mT; WT n=4, mock; mock/mT; A9$^{-/-}$ n=3, inf; mock; WT n=5, inf; mock; A9$^{-/-}$ n=6, inf; mT; WT n=9, inf; mT; A9$^{-/-}$ n=10). (**F**) Weight loss was monitored during infection. Percent weigh loss is normalized to starting weight. Each point represents the mean of all mice ±standard error (mock/mock; WT/A9$^{-/-}$ n=7, mock/mT; WT/A9$^{-/-}$ n=8, inf/mock; WT n=13, inf/mock; A9$^{-/-}$ n=15, inf/mT; WT n=19, inf/mT; A9$^{-/-}$ n=21). (**G**) 4 dpi, organs were homogenized and CFU was counted using spot plating (Limit of detection=LoD). Each point represents a single mouse (mock; mock/mT; WT n=4, mock; mock/mT; A9$^{-/-}$ n=3, inf; mock; WT n=5, inf; mock; A9$^{-/-}$ n=6, inf; mT; WT n=9, inf; mT; A9$^{-/-}$ n=10). (*A-B, E*) Vertical line on histogram is MFI. (*A-G*) Two-way ANOVA with Tukey multiple comparisons test (*$P \leq 0.05$, **$P \leq 0.01$, ***$P \leq 0.001$, ****$P \leq 0.0001$, ns=not significant).

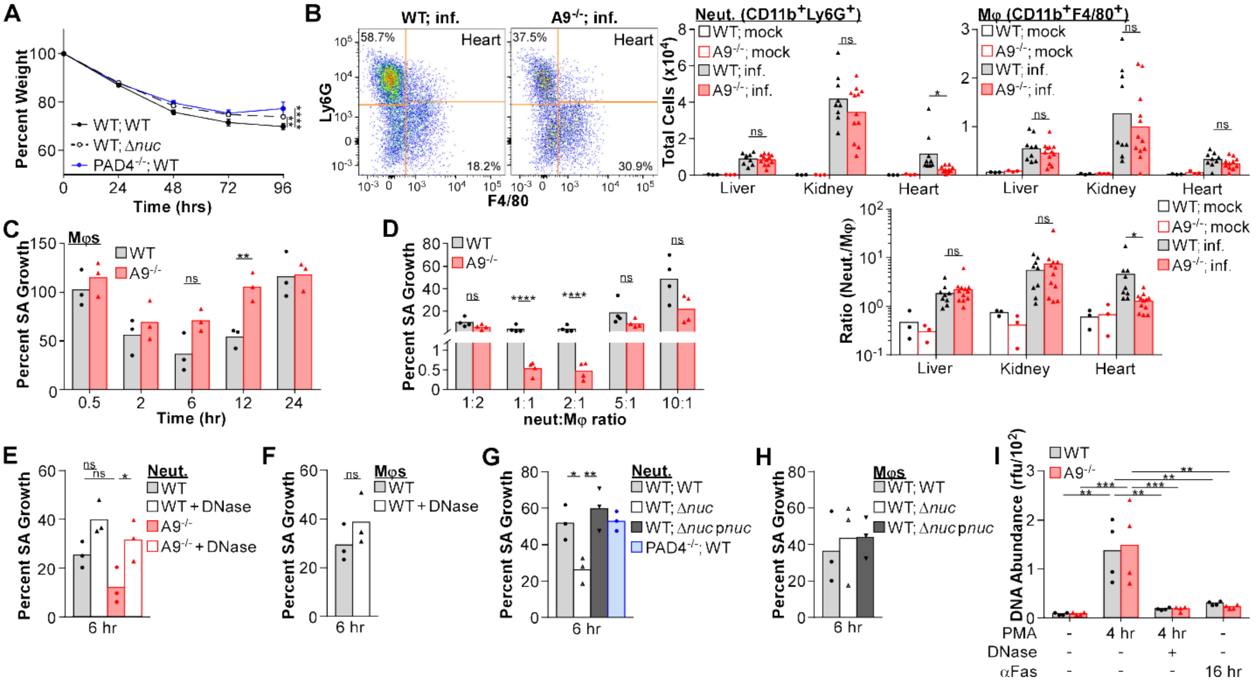

Fig. S6. NET formation minimally restricts *S. aureus* growth in the absence of Mφs. (**A-B**) Mice were systemically infected (inf.) with *S. aureus* (CFU=2x10^7) and (*A*) weight loss was monitored during infection. Percent weigh loss is normalized to starting weight. Each point represents mean of all mice ±standard error (WT; WT n=16, WT; Δ*nuc* n=20, PAD4$^{-/-}$; WT n=13). (*B*) Four dpi, organs were homogenized and the number of neutrophils and Mφs per 10^6 total events, and ratio of neutrophils to Mφs were quantified by flow cytometry. Each point represents a single mouse (mock n=3, WT; inf. n=9, A9$^{-/-}$; inf. n=12). (**C-H**), Immune cells were cultured with *S. aureus* (MOI=1). (*C, F, H*) Mφ, (*E, G*) neutrophil (Neut.), and (*D*) neutrophil-Mφ co-culture restriction of *S. aureus* growth was quantified by CFU spot plating. Percent growth of *S. aureus* (SA) calculated relative to *S. aureus* growth in the absence of immune cells. (*E-F*) Immune cells were cultured with *S. aureus* in the presence of DNase (8 U/mL). Each point represents the mean result (biological triplicate) of immune cells isolated from a single mouse (*C, E-H*, n=3; *D*, n=4). (**I**) Neutrophils were stimulated with PMA (100 nM) for 4 hr or αFas antibody for 16 hr and NET-like structures were isolated from the supernatants. DNase (8 U/mL) treatment occurred just after NET isolation. Isolated DNA was fluorescently stained and DNA abundance was quantified in a

plate reader. Each point represents the mean result (biological duplicate) of immune cells isolated from a single mouse (n=4). Two-way ANOVA with (*A-B, E, G-H*) Tukey or (*C-D, I*) Sidak's multiple comparisons test, or (*F*), unpaired *t*-test (*$P \leq 0.05$, **$P \leq 0.01$, ***$P \leq 0.001$, ****$P \leq 0.0001$, ns=not significant).

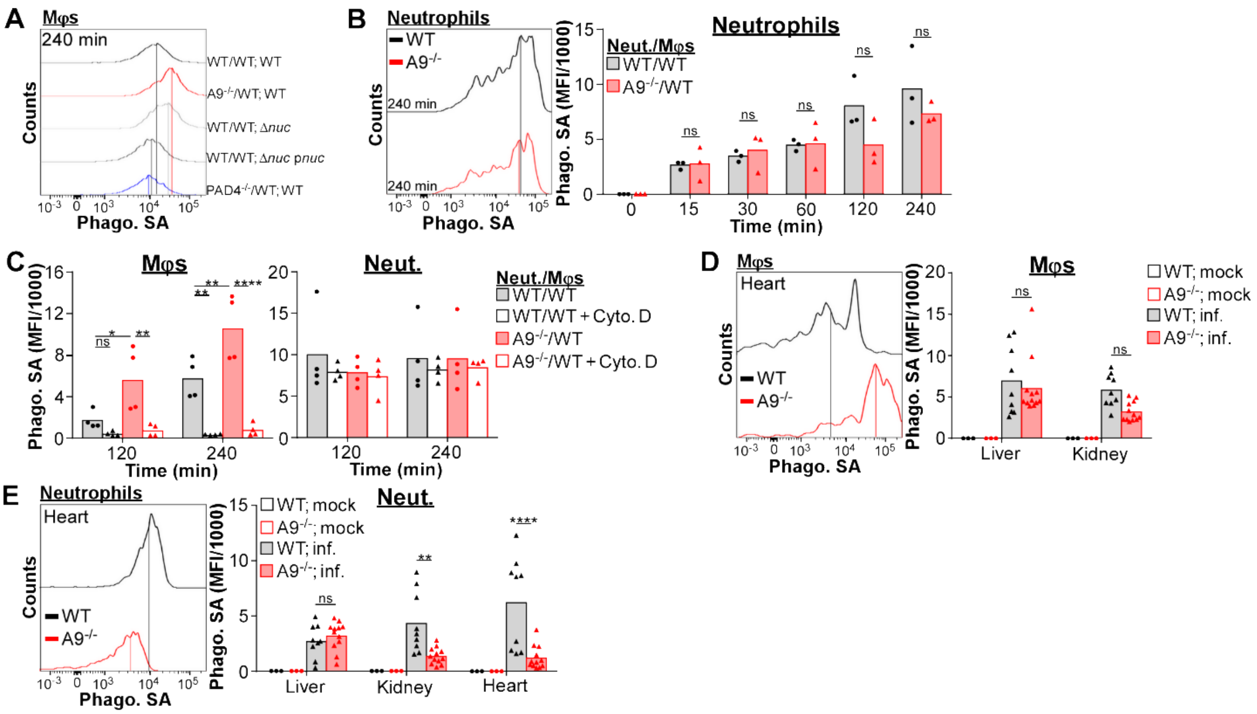

Fig. S7. Intracellular *S. aureus* levels are decreased in A9$^{-/-}$ neutrophils in during infection. (**A-C**) Neutrophil-Mφ co-cultures were stimulated with fluorescently labeled *S. aureus* (SA; MOI=10). (*A*) Representative histograms of phagocytosis (Phago.) by Mφs are provided. (*B-C*) phagocytosis by neutrophils and (*C*) Mφs was quantified by flow cytometry. (*C*) Mφs were pretreated with cytochalasin D (Cyto. D; 10 µg/mL) for 1 hr prior to adding neutrophils and *S. aureus*. (*A-C*) Background MFI subtracted from each time point. (*B-C*) Each point represents immune cells isolated from a single mouse (*B*, n=3; *C*, n=4). (**D-E**) Mice were systemically infected (inf.) with a fluorescent strain of *S. aureus* (p*SarA_sfGFP*, CFU=2x10^{7}). 4 dpi, organs were homogenized and the levels of *S. aureus* within (*D*) Mφs (CD11b^{+}F4/80^{+}Ly6G^{-}) and (*E*) neutrophils (CD11b^{+}Ly6G^{+}F4/80^{-}) in the tissues were quantified by flow cytometry. Background MFI from uninfected mice subtracted from infected. Each point represents a single mouse (mock n=3, WT; inf n=9, A9$^{-/-}$; inf n=12). (*A-B, D-E*) Vertical line on histogram is MFI. Two-way ANOVA with (*B*) Sidak's or (*C-E*) Tukey multiple comparisons test (**P*≤0.05, ***P*≤0.01, *****P*≤0.0001, ns=not significant).

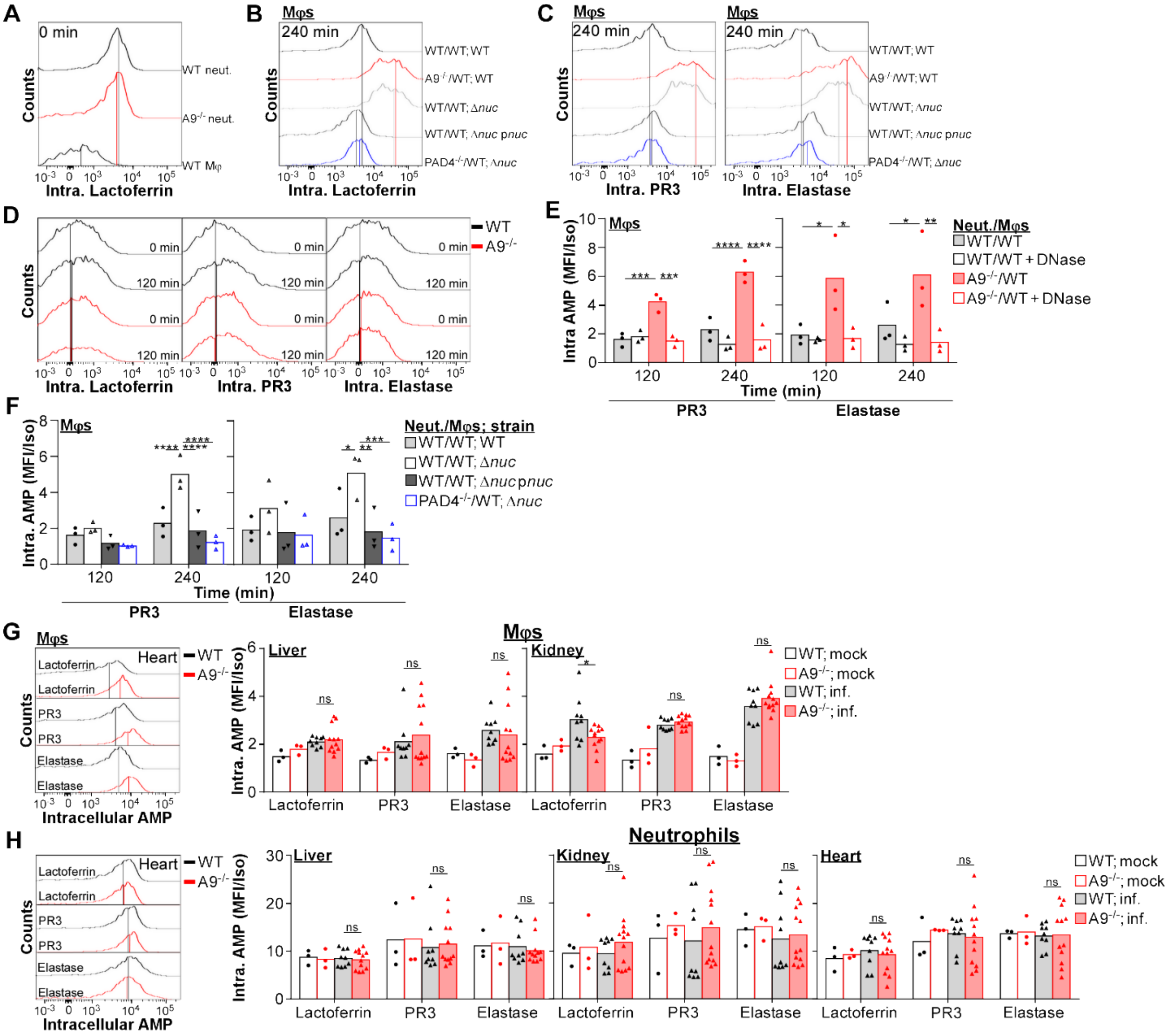

Fig. S8. Mφs do not express neutrophil specific AMPs. (**A**) Representative histograms of unstimulated neutrophils (Neut.) or Mφs stained for intracellular (Intra.) lactoferrin are provided. (**B-C, E-F**) Neutrophil-Mφ co-cultures or (**D**) Mφs alone were stimulated with *S. aureus* (MOI=10) and representative histograms for intracellular (*B, D*) lactoferrin, (*C-D*) PR3, and neutrophil elastase are provided. (*E-F*) The intracellular abundance of PR3 and neutrophil elastase was quantified by flow cytometry. (*E*) Immune cells were cultured with *S. aureus* in the presence of DNase (8 U/mL). MFI normalized by an isotype control. (*E-F*) Each point represents immune cells isolated from a single mouse (n=3). (**G-H**) Mice were systemically infected (inf.) with a fluorescent strain of *S. aureus* (p*SarA_sfGFP*, CFU=2x10^{7}). 4 dpi, organs were homogenized and the

intracellular levels of lactoferrin, PR3, and elastase in (*G*) Mφs and (*H*) neutrophils in the tissues were quantified by flow cytometry. MFI normalized by an isotype control. Each point represents a single mouse (mock n=3, WT; inf n=9, A9$^{-/-}$; inf n=12). (*A-D, G-H*) Vertical line on histogram is MFI. (*E-H*) Two-way ANOVA with Tukey multiple comparisons test (*$P \leq 0.05$, **$P \leq 0.01$, ***$P \leq 0.001$, ****$P \leq 0.0001$, ns=not significant).

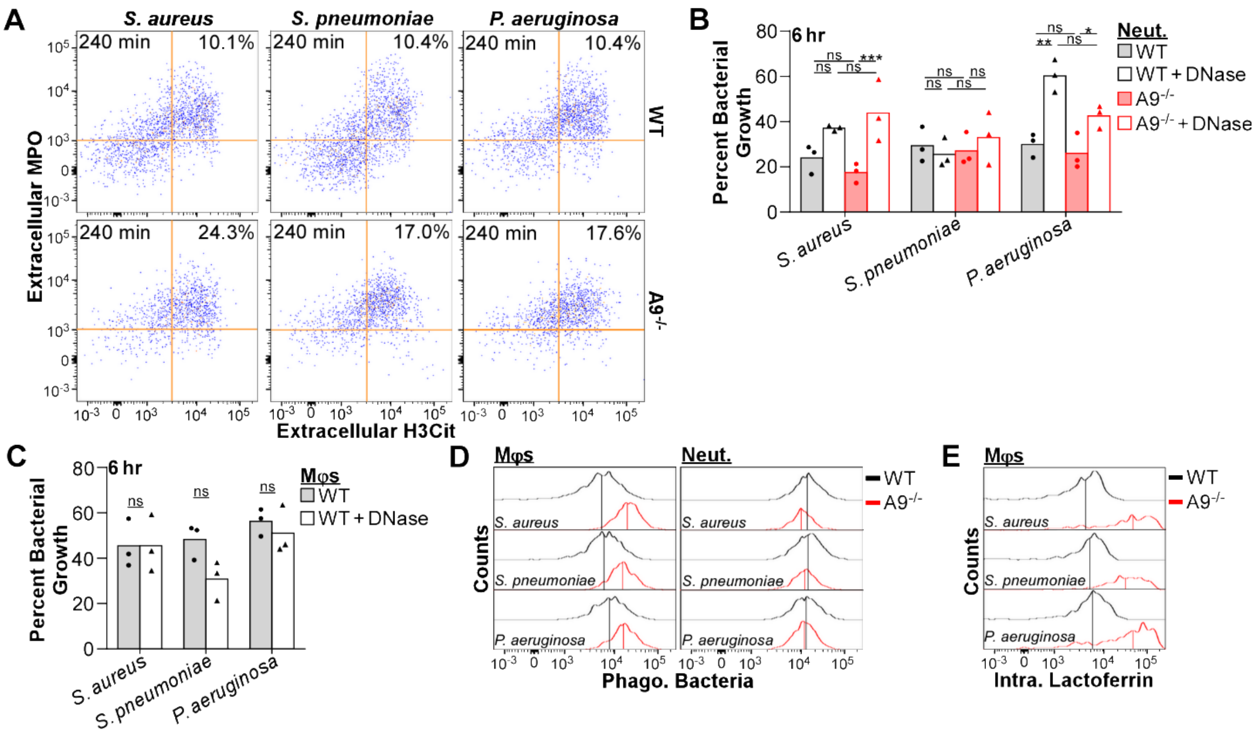

Fig. S9. NET formation alone restricts growth *P. aeruginosa* but not *S. pneumoniae*. (**A-E**) Immune cells were cultured with *S. aureus*, *S. pneumoniae*, and *P. aeruginosa* (*A, D-E*, MOI=10; *B-C*, MOI=1). (*A*) The percentage of neutrophils ($Ly6G^+CD11b^+$) undergoing suicidal NETosis (Dead: extracellular $dsDNA^+MPO^+H3Cit^+$) was quantified by flow cytometry. (*B*) Neutrophil (Neut.) or (*C*) Mφ restriction of bacterial growth was quantified by CFU spot plating. Percent bacterial growth calculated relative to bacterial growth in the absence of immune cells. (*B-C*) Immune cells were cultured with bacteria in the presence of DNase (8 U/mL). Neutrophil-Mφ co-cultures (ratio=1:1) were stimulated with fluorescently labeled bacteria and (*D*) phagocytosis of bacteria by Mφs or neutrophils, and (*E*) the level of intracellular lactoferrin in Mφs was quantified by flow cytometry. (*B-C*) Each point represents the mean result (biological triplicate) of immune cells isolated from a single mouse (n=3). (*D-E*) Vertical line on histogram is MFI. Two-way ANOVA with (*B*) Tukey or (*C*) Sidak's multiple comparison test (*$P \le 0.05$, **$P \le 0.01$, ***$P \le 0.001$, ns=not significant).
